# Multiplexed Delivery of Synthetic (Un)Conjugatable Ubiquitin and SUMO2 Enables Simultaneous Monitoring of Their Localization and Function in Live Cells

**DOI:** 10.1002/cbic.202200122

**Published:** 2022-03-11

**Authors:** Guy Mann, Pradeep Sadhu, Ashraf Brik

**Affiliations:** ^1^ Schulich Faculty of Chemistry Technion-Israel Institute of Technology Haifa 3200008 Israel

**Keywords:** chemical protein synthesis, fluorescent probes, post-translational modifications, protein delivery, protein modifications

## Abstract

Ubiquitin (Ub) and its related small Ub like modifier (SUMO) are among the most influential protein post‐translational modifications in eukaryotes. Unfortunately, visualizing these modifications in live cells is a challenging task. Chemical protein synthesis offers great opportunities in studying and further understanding Ub and SUMO biology. Nevertheless, the low cell permeability of proteins limits these studies mainly for *in vitro* applications. Here, we introduce a multiplexed protein cell delivery approach, termed MBL (multiplexed bead loading), for simultaneous loading of up to four differentially labeled proteins with organic fluorophores. We applied MBL to visualize ubiquitination and SUMOylation events in live and untransfected cells without fluorescent protein tags or perturbation to their endogenous levels. Our study reveals unprecedented involvements of Ub and SUMO2 in lysosomes depending on conjugation states. We envision that this approach will improve our understanding of dynamic cellular processes such as formation and disassembly of membraneless organelles.

## Graphical abstract

**Live cell imaging** of post‐translationally modified proteins is key to understanding their involvement in both healthy and disease states. We report multiplexed bead loading (MBL) for the simultaneous delivery of several synthetic proteins to the same cells. Using MBL, we reveal unknown involvements of ubiquitin and SUMO2 in lysosomes.

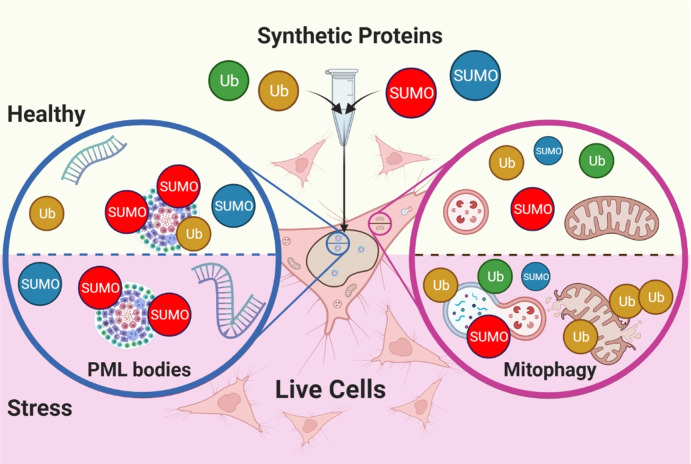

## Introduction

Post‐translational modifications (PTMs) of proteins substantially expand their functional diversity and their misregulation is correlated with various pathological states.^[1]^ The dynamic nature of PTMs introduces substantial challenges when imaging their incorporation and removal in real‐time.^[2]^ Moreover, imaging small protein modifiers such as ubiquitin (Ub) and Ub like modifiers (UBLs) requires their fusion to fluorescent proteins (e. g. GFP), which are significantly larger than these PTMs and could result in various artifacts.[^3^, ^4^, ^5^, ^6^] Despite the power of molecular biology approaches to express proteins bearing various peptides and proteins tags, these are still limited in generating modified proteins and allow simultaneous tracking of their cellular functions in real time, without affecting their properties.^[7]^

The need to access site‐specifically modified proteins resulted in substantial advancements in chemical and semi‐protein synthesis for the preparation of uniquely modified proteins.^[8]^ Often, these synthetic proteins are “custom‐made” to answer fundamental biological questions by including elements that are often inaccessible by recombinant expression. Due to various challenges, synthetic proteins are prepared in a relatively small scale and are often impermeable, limiting their studies in live cells. On the other hand, various methods to deliver proteins have been developed and optimized for specific protein targets.^[9]^ Therefore, finding the most effective method for delivering uniquely modified synthetic proteins for biological studies is still challenging and requires further development.

As of today, transiently attaching a protein cargo to a cell penetrating peptide (CPP) is the most efficient and therapeutically relevant approach for protein cell delivery.^[10]^ Despite its usefulness, CPP mediated delivery proceeds through endocytic mechanism that mostly results in high background noise due to endosomal entrapment, which forces extensive optimization for each protein cargo.^[11]^ In addition, the strong cargo dependence of CPP delivery, generates a substantial bias for comparison between two different cargoes delivered by the same CPP.^[12]^ In many cases, cytosolic delivery can be insufficient, requiring significant time and resources to prepare the desired proteins, without guaranteeing sufficient delivery required for measurable biological effect(s).

On the other hand, physical methods which are used to transiently disrupt the cell membrane, can deliver proteins to live cells with minimal cargo bias. Unfortunately, many of these methods require high specialty, produce limited number of loaded cells and suffer from toxicity.^[13]^ To best of our knowledge, the most simple, mild and cost effective physical method utilizes glass beads to enable direct cytosolic delivery in a process termed bead loading (BL).^[14]^ Using this method, various groups have reported successful delivery of synthetic peptides to achieve live cell protein engineering,[^15^, ^16^] catalytic installation of PTMs to histones^[17]^ imaging of histone PTMs^[18]^ and mRNA translation by ribosomes.^[19]^ Importantly, treated cells exhibited normal proliferation rate, emphasizing the low toxicity generated by loading cells using this approach.^[20]^

In order to unleash the full power of protein synthesis for studies in a cellular environment, we feel that the glass ceiling of delivering these proteins could be breached using BL. We therefore aimed to expand the limit of this approach to deliver and study multiple synthetic protein probes in same cells. Herein, we report on a powerful multiplexed bead loading approach (MBL) that is independent of CPP and genetic manipulation, for the simultaneous imaging of up to four differentially labeled synthetic proteins with organic fluorophores. Our results provide unprecedented view on the localization and function of conjugated and unconjugated Ub and the small Ub like modifier (SUMO) isoform 2 (SUMO2), in live cells.

## Results and Discussion

To examine the applicability of MBL to deliver multiple synthetic proteins, we chose Ub and SUMO2 as model synthetic proteins for live cell delivery to shed light on their localization and function under different conditions. We selected these two proteins for the following reasons; 1) The challenges in their live cell imaging as ectopically expressed GFP fused forms. 2) Their distinct cellular distributions and drastic changes in different conditions. 3) Their crosstalk and functional overlaps in many biological pathways, 4) Straightforward synthesis of both proteins and their analogues.[^21^, ^22^]

Ubiquitination, which proceed through the attachment of C‐terminal glycine 76 (Gly76) of Ub to a specific lysine in a protein substrate was originally discovered to induce the substrates degradation.^[23]^ This PTM also regulates several other cellular processes by conjugating Ub units to one of its seven lysines or N‐terminus, generating polyUb chains with different signaling depending on chain topology.^[24]^ In mammalian cells, SUMO is mainly involved in regulating nuclear processes and exists in three major isoforms (SUMO1‐3).^[25]^ SUMO1‐3 mostly localize to membraneless organelles (MLOs) formed by liquid‐liquid phase separation (LLPS), such as PML bodies, comprising of the promyelocytic leukemia (PML) protein.^[26]^ Notably, Ub and SUMO1‐3 share many substrates where they compete for the same modification sites.^[27]^ The complex relationship between SUMO2 and Ub is emphasized in their conjugation to PML *via* SUMO dependent ubiquitination machinery (StUB), where RNF4 functions as a SUMO targeted Ub ligase (StUBL).^[26]^

### Chemical synthesis of Ub and SUMO2 probes

**Figure 1:**
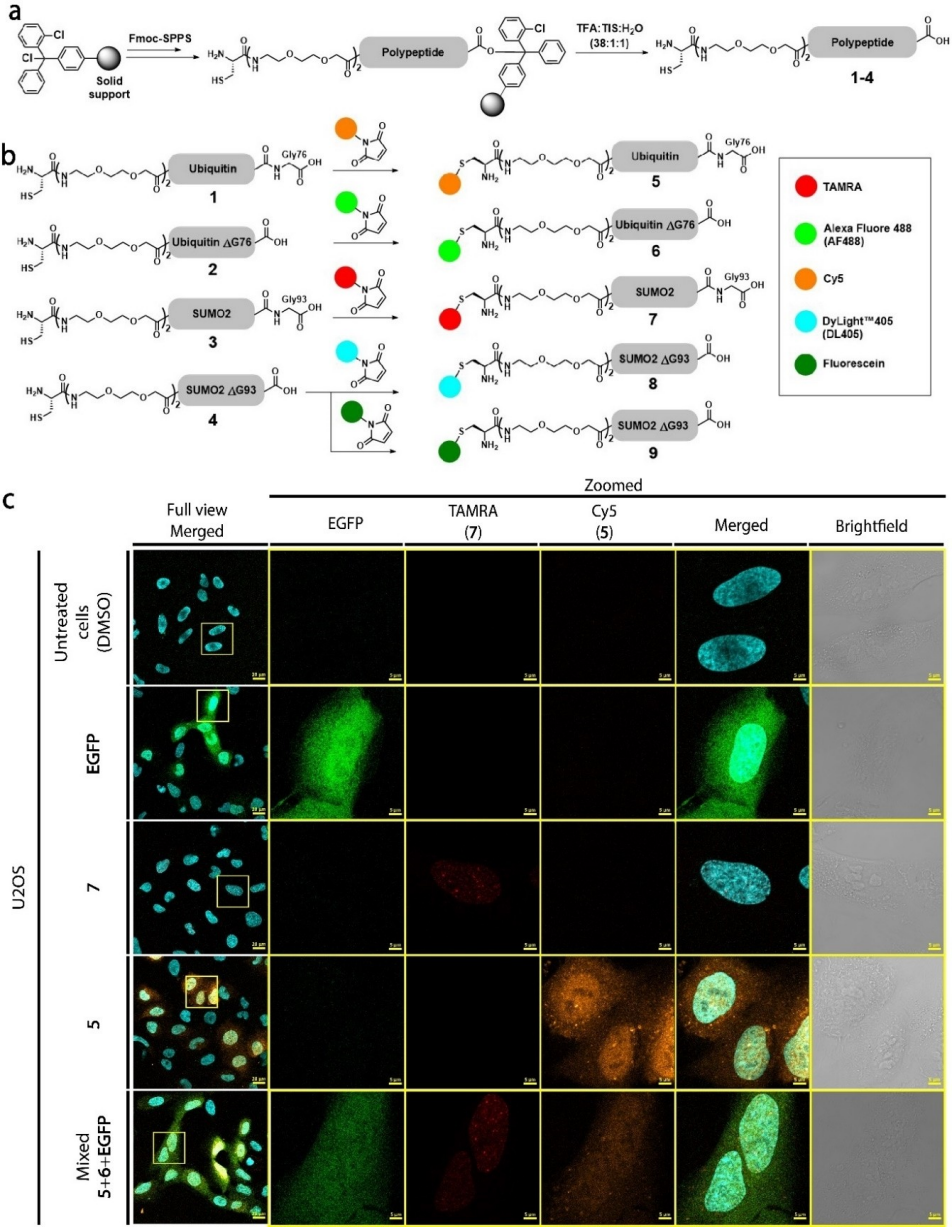
Synthesis and MBL of fluorescent proteins. (a) Fmoc‐SPPS of **1**–**4** on a 2‐CTC resin. (b) Labeling of **1**–**4** with maleimide dyes to generate probes **5**–**9**. (c) Live cell LSCM images of U2OS cells loaded with EGFP (green) **5** (gold), **7** (red) and Hoechst nuclear stain (Cyan). Full view scale bars are 20 μm. Zoomed scale bars are 5 μm.

Employing Fmoc‐SPPS on 2‐chlorotrityl chloride (2‐CTC) resin, we prepared Ub‐COOH (**1**) to enable conjugation to its cellular substrates (Figure 1a, Supporting Information Figure S1). To probe the effect of Ub's conjugation in cellular processes, we also prepared its unconjugatable form by deleting Gly76 (**2**, UbΔG76) (Figure 1a, Supporting Information Figure S2). Our probes also included N‐terminal cysteine (Cys), separated *via* a flexible linker from Ub sequence, to allow labeling with the organic fluorophore *via* maleimide chemistry. Similarly, we synthesized SUMO2 with Cys48Ala mutation (**3**) to allow specific labeling with the N‐terminal Cys (Figure 1a, Supporting Information FigureS3). This mutation was reported not to affect SUMO2 secondary structure, thermal stability and conjugation.^[28]^ We also prepared unconjugatable SUMO2 by deleting its Gly93^[29]^ (**4**, SUMO2ΔG93) (Figure 1a, Supporting Information Figure S4). Following their HPLC purification, we labeled analogues **1**–**4**, by reacting the N‐terminal Cys with maleimide dyes to generate probes **5**–**8** (Figure 1b, Supporting Information Figures S1–S4). Since DL405 dye is not compatible with the live cell nuclear stain Hoechst, lysosomal stain lysotracker blue (LTB) and fluorescent gel imaging, we used construct **4** to generate an additional fluorescein tagged derivative; probe **9** (Figure 1b, Supporting Information Figure S4).

### Live cell protein delivery

With Ub **5** and SUMO2 **7** probes in hand, we turned our attention for simultaneous live cell delivery. We used low micromolar concentration of these probes to avoid substantial effects on the endogenous Ub and SUMO2 concentrations, which are estimated to be in ∼80‐100 micromolar range.[^30^, ^31^] We mixed probes Ub **5** and SUMO2 **7** with the commercial enhanced green fluorescent protein (EGFP) in PBS, containing the non‐ionic surfactant pluronic™ F‐68. We included EGFP to test the efficacy of MBL with larger proteins and to compare its distribution to Ub and SUMO2. We loaded mixed and separate proteins to U2OS cells at 3‐μM final concentration and two minutes of incubation (see general procedures in the Supporting Information). Gratifyingly, we confirmed successful delivery by laser scanning confocal microscope (LSCM) (Figure 1c). As expected, EGFP was mostly diffused thought the cells and excluded from membranous organelles (e. g. mitochondria). Ub **5** was diffused through the cell with certain preference to cytosolic vesicles and was excluded from nucleoli.^[22]^ SUMO2 **7** was also distributed, as previously reported, to distinct nuclear puncta.^[32]^

Interactions between organic dyes and biomolecules inside cells can potentially affect the subcellular localization of the free dye.^[33]^ To confirm that the distribution of our synthetic probes is unaffected by fluorescent dye, we conjugated our synthetic Ub **1** with fluorescein‐maleimide and TAMRA‐maleimide generating Ub probes **10** and **11** (Supporting Information Figure S1). LSCM live cell imaging of Ub probes **5**, **10** and **11** in same cells confirmed that the nature of fluorescent dye had no visible effect on the cellular distribution of Ub (Supporting Information Figure S6).

We then aimed to simultaneously deliver three different synthetic proteins. We used Ub **5**, SUMO2 **7** and the FITC‐labeled TAB2‐ZnF4 **12** (Supporting Information Figure S5), which was reported to bind Lys63 polyUb chains when ectopically expressed with GFP tag.^[34]^ Mixing these proteins at 3‐μM concentration for each protein resulted in their successful delivery to live U2OS cells (Supporting Information Figure S7). In order to confirm that probes **5** and **7** can be conjugated to cellular proteins, we treated U2OS cells having Ub **5**, SUMO2 **7** and ZnF4 **12** with the proteasome inhibitor MG132. We then lysed these cells and performed fluorescent gel analysis, which confirmed that both Ub **5** and SUMO2 **7** are functional and form the expected high molecular weight conjugates (Supporting Information Figure S8).

**Figure 2:**
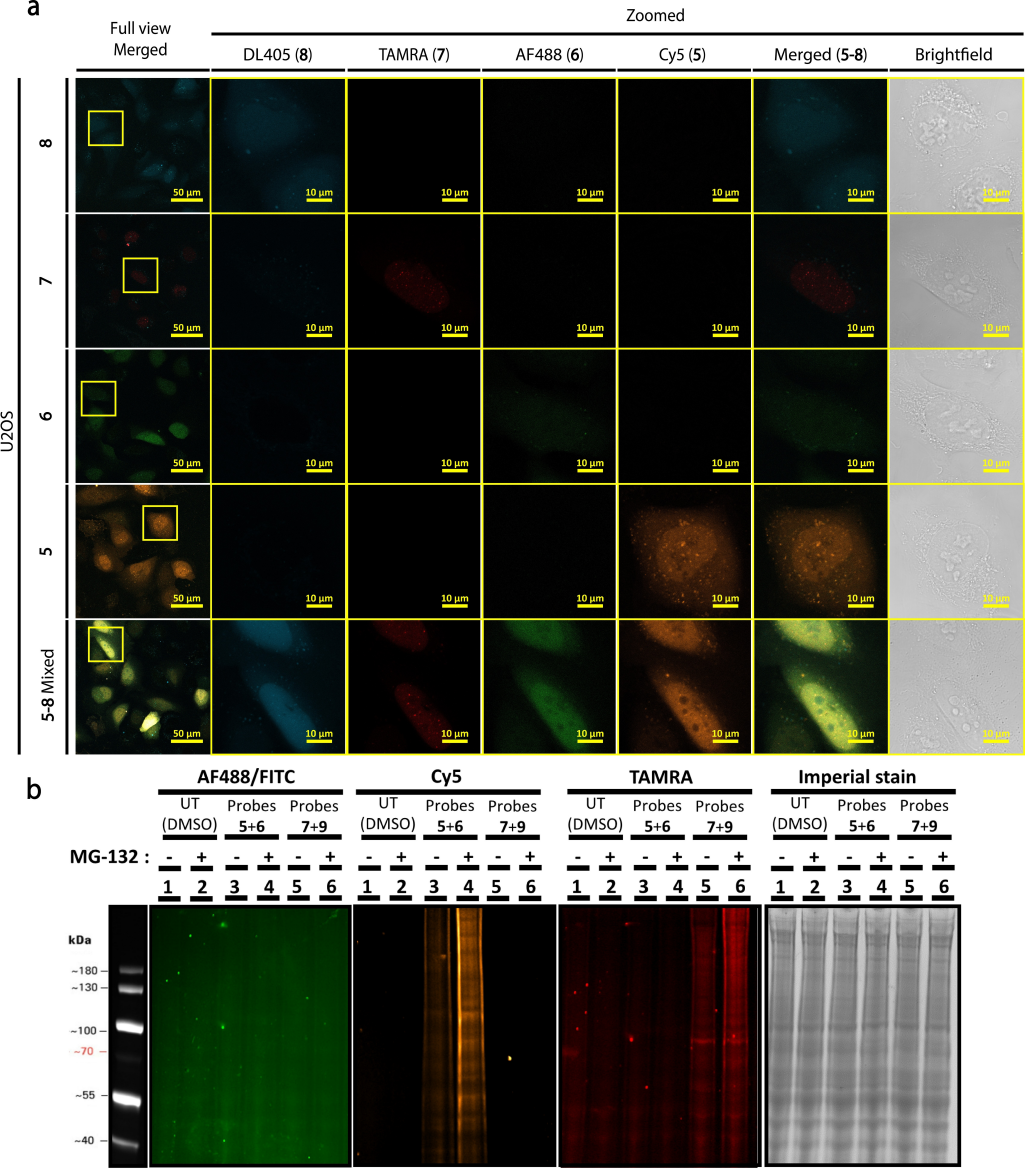
MBL of four synthetic proteins in live U2OS cells. (a) Zoomed LSCM images of U2OS cells loaded with single and multiplexed probes **5**–**9**. Scale bars are 10 μm. Full view scale bar is 50 μm. (b) Fluorescent gel imaging of U2OS lysate treated with (lanes 2, 4, 6) and without (lanes 1, 3, 5) MG132. Lanes 1 and 2 are loaded without probes. Lanes 2 and 3 are with Ub probes **5** and **6**. Lanes 4 and 5 are loaded with SUMO2 probes **7** and **9**. Loading control is imperial stain. AF488 channel was imaged using maximum exposure.

Fascinated by the contrast between SUMO2 and Ub live cell distribution, we questioned whether this is determined by their conjugation to cellular targets or by protein‐protein interactions. Therefore, we performed MBL of the four synthetic proteins to live cells, including Ub **5** and SUMO2 **7** and their unconjugatable forms, **6** and **8**, at final concentration of 2 μM for each protein (Figure 2a). As a spectral separation control between the fluorescent dyes, we included single stained cells loaded separately with the same concentration of each probe. Under these conditions, we did not observe bleed‐through between these fluorescent dyes, allowing us to use them for subsequent experiments (Figure 2b). Notably, in this experiment we visualized a strong contrast in nuclear localization between SUMO2 probes **7** and **8**, suggesting that SUMO2 puncta in the nucleus requires its conjugation. This is further supported by fluorescent gel analysis, where the C‐terminal Gly was critical in forming high molecular weight conjugates when cells were loaded with Ub **5** and **6** or SUMO2 **7** and **9** (Figure 2b).

### Live cell imaging of synthetic probes reveals their localization and functional differences

We then turned to probe Ub and SUMO2 behaviors in both healthy and stressed conditions. For this, we chose the degradation of damaged mitochondria *via* PINK1/parkin mediated mitophagy as an example for Ub mediated process.^[35]^ In mitophagy, retention and stabilization of PTEN‐induced kinase 1 (PINK1) on the mitochondrial outer membrane (MOM) functions as a mitochondrial damage sensor. Upon mitochondrial damage, PINK1 phosphorylates serine 65 in polyUb conjugates (poly(p)Ub) attached to MOM proteins. Generation of poly(p)Ub results in the recruitment of RBR E3 ubiquitin‐ligase; parkin. Parkin is further activated by PINK1 phosphorylation resulting in rapid amplification of the poly(p)Ub content and global changes in ubiquitination landscape.^[36]^ This mitochondrial poly(p)Ub coat recruits autophagy components such as LAMP1, NBR1 and P62 to shuttle damaged mitochondria to mitolysosomes for degradation.^[36]^ PINK1/parkin mediated mitophagy is completely dependent of parkin and does not occur in cells lacking parkin expression such as U2OS cells. In order to induce parkin/PINK1 dependent mitophagy, most studies utilized global mitochondrial depolarization by treating cells with the protonophore carbonyl cyanide 3‐chlorophenylhydrazone (CCCP).^[37]^

**Figure 3:**
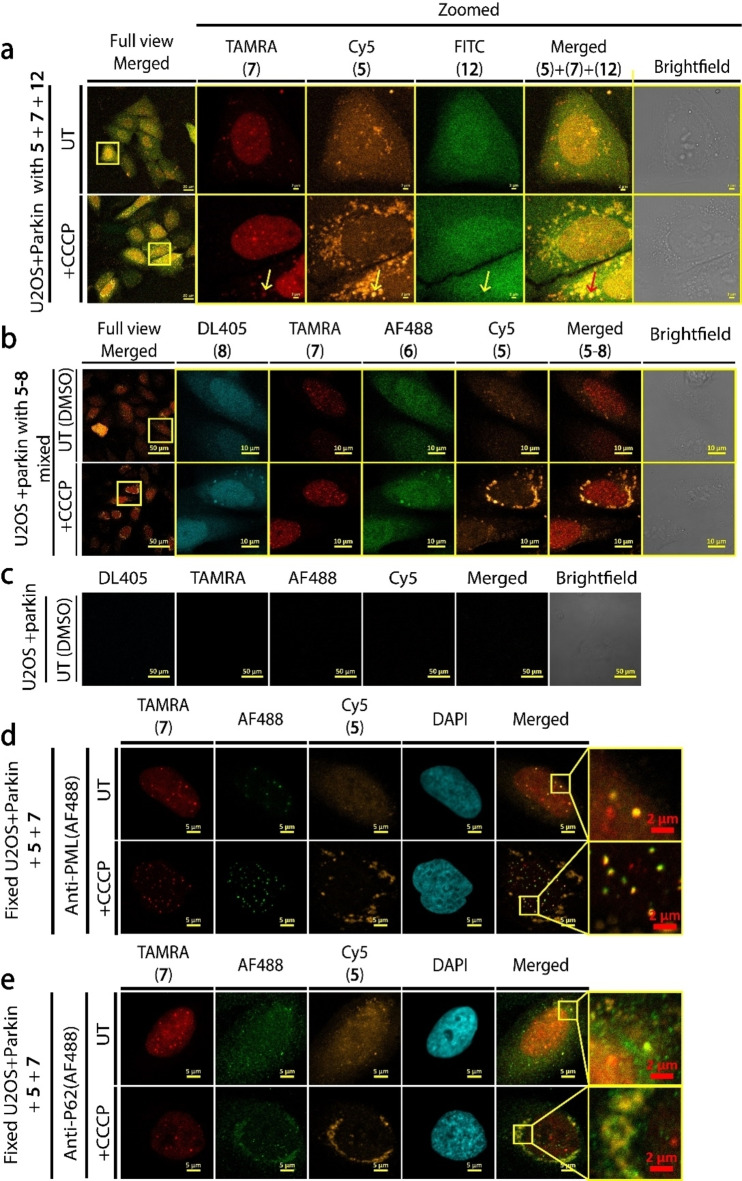
Multiplexed protein delivery to U2OS +parkin cells with and without CCCP treatment. (a) Full view and zoomed LSCM images of live cells loaded with Ub **5**, SUMO2 **7** and ZnF4 **12**. Scale bars are 10 μm. Full view insert scale bar is 50 μm (b) Full view and zoomed LSCM of live cells loaded with multiplexed probes **5**–**8**. Scale bars are 50 μm for full view and 10 μm for zoomed images. (c) Auto fluorescence control of live U2OS +parkin cells from B loaded without probes (beads and DMSO). Scale bars are 50 μm. (d) LSCM imaging of **5** (gold) and **7** (red) and anti‐PML antibody (green) in fixed cells. (e) LSCM imaging of **5** (gold) and **7** (red) and anti‐P62 antibody (green) in fixed cells. Scale are 5 μm (yellow bar) and 2 μm (red bars).

In order to demonstrate the incorporation of synthetic Ub **5** into poly(p)Ub chains by changes in its cellular distribution, we loaded Ub **5**, SUMO2 **7**, and ZnF4 **12** to U2OS cells expressing untagged human parkin (U2OS+parkin)[^38^, ^39^] and induced mitophagy by CCCP. Gratifyingly, we observed the reported global recruitment of Ub probe **5** to damaged mitochondria at the perinuclear area (Figure 3a).^[40]^ Surprisingly, in addition to the reported distribution in the nucleus of SUMO2, it was also localized to cytosolic puncta that increased both in intensity and size upon CCCP treatment. These puncta did not contain the diffused FITC‐ZnF4 **12**, suggesting that these are membranous vesicles. We could not detect correlation between probes Ub **5** or SUMO2 **7** and probe **12** with or without CCCP treatment, we therefore excluded probe **12** from our remaining experiments (Figure 3a).

To compare the functional difference between the free and conjugated forms of Ub and SUMO2 in stress, we loaded probes **5**–**8** into U2OS+parkin cells and treated them with CCCP (Figure 3b). While Ub **5** and SUMO2 **7** localized as we observed previously, we identified an unexpected increase in the localization of unconjugatable Ub **6** to vesicles. Despite the success in delivering four proteins, the low brightness of the DL405 dye in probe **8** resulted in fluorescence bleed‐trough from puncta in the AF488 channel in CCCP treated cells. Therefore, we used probe **9** instead of **8** as unconjugatable SUMO2 in our following experiments (Figure 3b and c).

To further expand our mechanistic understanding on the functions of Ub **5** and SUMO2 **7** in U2OS+parkin cells, we loaded cells with these probes followed by CCCP treatment, fixation and immunofluorescence analyses with organelle markers. We used the antibodies for mitochondrial matrix protein MTCO2, lysosomal marker LAMP1 and for the phosphorylated Ub at Ser65 as a mitophagy marker (pUb).^[41]^ LSCM and super resolution microscopy (SRM) *via* structure illuminated microscopy (SIM^2^) confirmed that Ub **5** is indeed recruited to damaged mitochondrial sites during mitophagy in a CCCP dependent manner (Supporting Information Figure S9). In contrast to live cells, in fixed cells we could not visualize SUMO2 localization to cytosolic vesicles or mitochondria, which is likely due to vesicle disruption during our cell fixation procedure.[^9^, ^42^]

During parkin/PINK1 mitophagy, P62 aggregates damaged mitochondria in the perinuclear region *via* its PB1 domain mediated polymerization to generate polyubiquitinated aggregates.^[43]^ Subsequently, this results in recruitment of LC3 autophagy mediator for transporting these aggregates to autophagosomes.^[44]^ In addition to P62, this process involves another autophagy adaptor NBR1.^[45]^ Interestingly, P62 also localizes to PML bodies under certain conditions^[46]^ and is involved in nuclear degradation of ubiquitinated proteins.^[47]^ In addition to being the canonical substrate of SUMO2/3, PML is a crucial component of MLOs termed PML bodies.^[32]^

To investigate the involvement of SUMO2 **7** and Ub **5** in MLOs, we loaded them to live U2OS+parkin cells following treatment with and without CCCP. After, these cells were fixed and stained for immunofluorescence analyses with antibodies for PML and the autophagy adaptors P62/SQSTM1/Sequestosome‐1 and NBR1.^[48]^ LSCM images confirmed that SUMO **7** is localized to PML in MLOs, in contrast to Ub probe **5**, which did not show significant PML localization in both CCCP treated and untreated cells (Figure 3d). Under these conditions, P62 overlapped with Ub **5** in cytosolic vesicles in untreated cells and in perinuclear mitochondrial aggregates induced by CCCP treatment (Figure 3e). Similar results were obtained with anti‐NBR1 staining (Supporting Information Figure S10). In our hands, P62 did not localize to SUMO2 positive nuclear bodies under both conditions, but this requires further examination. As in our previous experiments, we could not identify SUMO2 **7** in cytosolic vesicles in fixed cells.

### UbΔG76 is recruited to lysosomes

**Figure 4:**
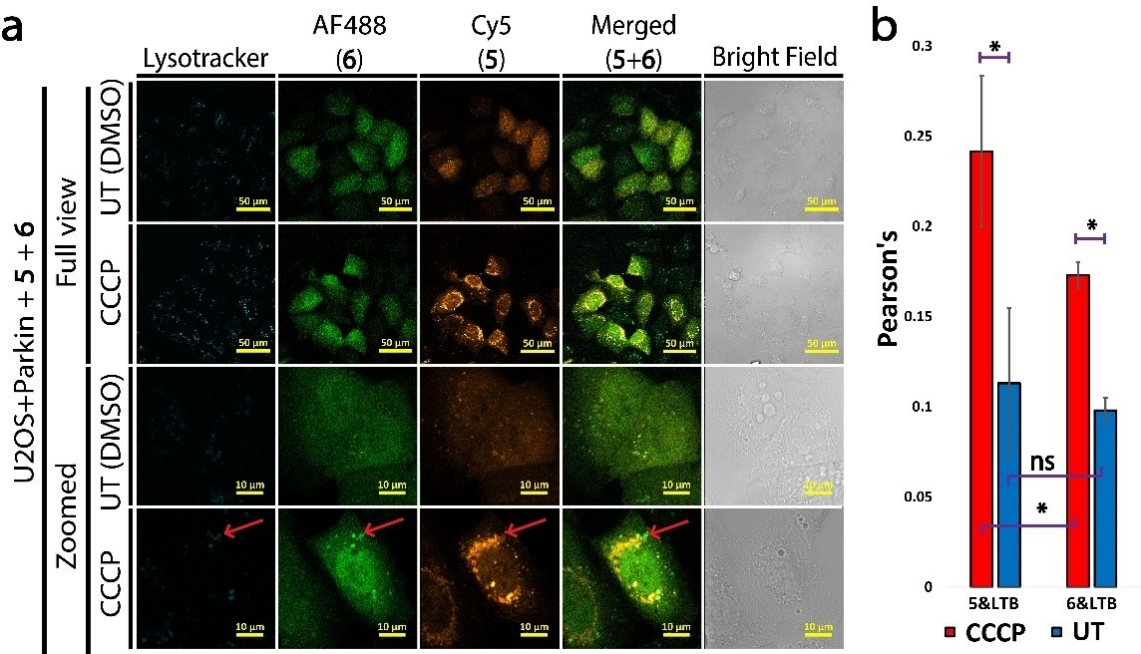
Multiplexed loading of Ub **5** and **6** to live U2OS+parkin cells with and without CCCP treatment. (a) Full view and zoomed LSCM images with LTB as lysosome stain. (b) Colocalization analysis of cells from A using averaged Pearson's coefficient from three independent experiments (>100 cells each). Ub probe fluorescence was used for ROI. Scale bars are 50 μm for full view and 10 μm for zoomed images. * P‐Value is below 0.01, ** P‐Value is below 0.05, ns is not significant. Error bars are standard deviation.

In our previous experiments we observed localization of UbΔG76 (**6**) cytosolic vesicles mainly upon CCCP treatment, which has not been reported before. To further test this, we delivered Ub **5** and **6** analogues into U2OS+parkin cells and examined the nature of these vesicles using the lysosome stain lysotracker blue (LTB) after CCCP treatment. These results, confirmed that both Ub **5** and **6** are recruited to lysosomes (Figure 4), which suggests a possible functional role of unconjugated Ub in live cells, yet in an unclear mechanism.

### SUMO2 is conjugated to lysosomes

**Figure 5:**
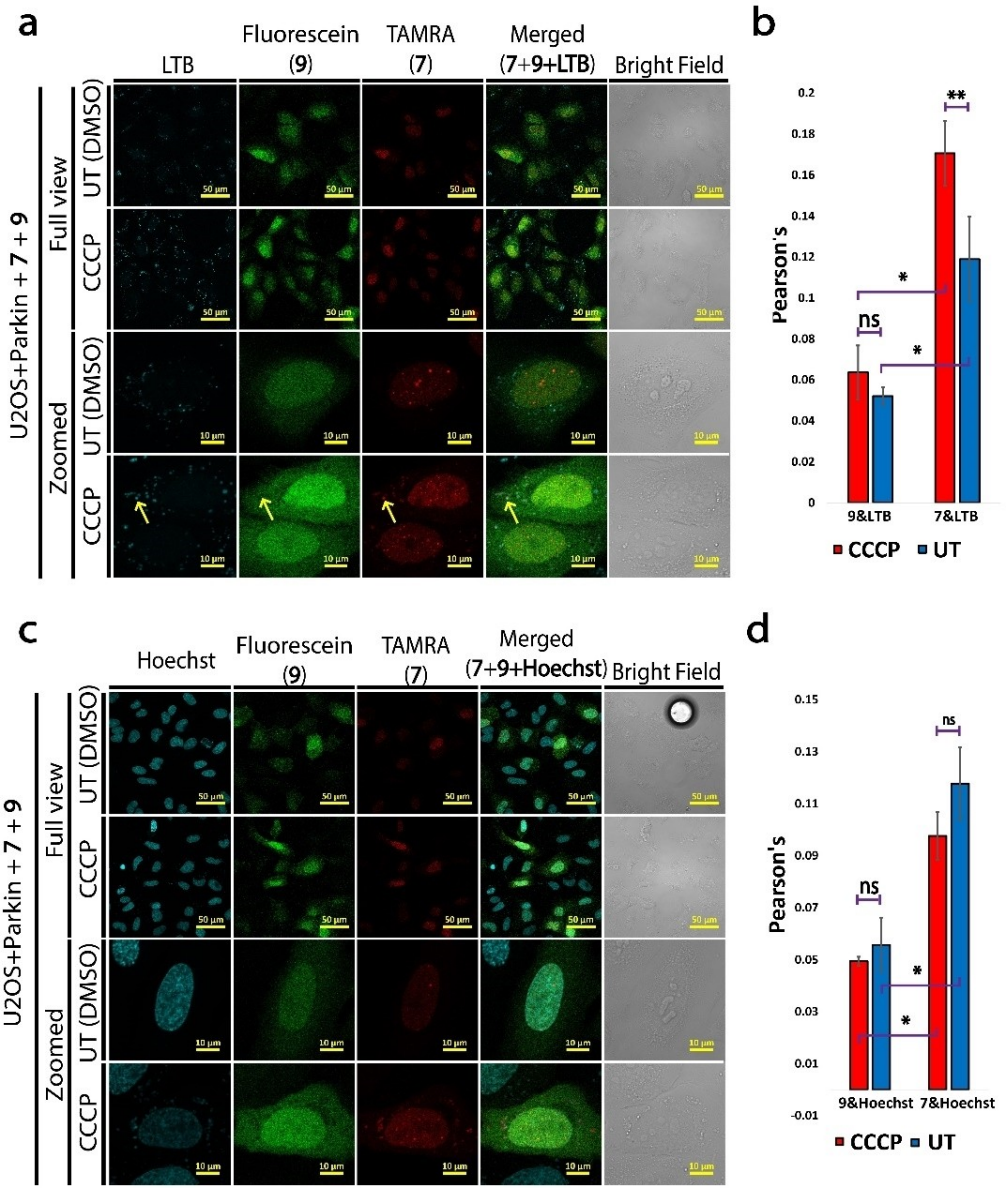
Multiplexed loading of SUMO2 probes **7** and **9** to live U2OS+parkin cells with and without CCCP treatment. (a) Full view and zoomed LSCM images with **7** (red) and **9** (green) and LTB (cyan) as lysosome stain. (b) Colocalization analysis of cells from A using averaged Pearson's coefficient from three independent experiments (>100 cells each). LTB fluorescence was used for ROI. (c) Full view and zoomed LSCM images of **7** (red) and **9** (green) and Hoechst (cyan) as a nuclear stain. (d) Colocalization analysis of cells from C using averaged Pearson's coefficient from three independent experiments (>100 cells each). Hoechst fluorescence was used for ROI. * P‐Value is below 0.01, ** P‐Value is below 0.05, ns is not significant. Error bars are standard deviation.

As of today, there are only a few reports on the role of SUMO2 in mitophagy. On the other hand, several mitochondrial proteins are reported to undergo SUMO2/3 conjugation, suggesting a possible role for SUMO2 in mitochondrial maintenance.^[49]^ Building on SUMO2’s localization to cytosolic vesicles in our previous experiments, we examined how SUMO2 conjugation is involved in this process. For this, we delivered SUMO **7** and **9** probes into live U2OS +parkin cells, followed by CCCP treatment and staining with LTB. LSCM imaging confirmed that SUMO2 **7** is localized to lysosomes, suggesting its involvement in a late stage of mitophagy (Figure 5a and b). On the other hand, SUMO2ΔG93 **9** was excluded from lysosomes in both CCCP treated and untreated cells (Figure 5a and b). To the best of our knowledge, SUMO2 conjugation to lysosomes in both basal and parkin mediated mitophagy was never reported. Confocal microscopy revealed that SUMO2 **7** nuclear localization was not significantly affected by CCCP treatment, as evident from co‐localization with the DNA dye Hoechst. However, this co‐localization is affected by SUMO2’s availability for conjugation since SUMOΔG93 **9** showed a significant reduction in its nuclear localization (Figure 5c and d).

### SUMO2 is conjugated to autophagosomes in mitophagy

**Figure 6:**
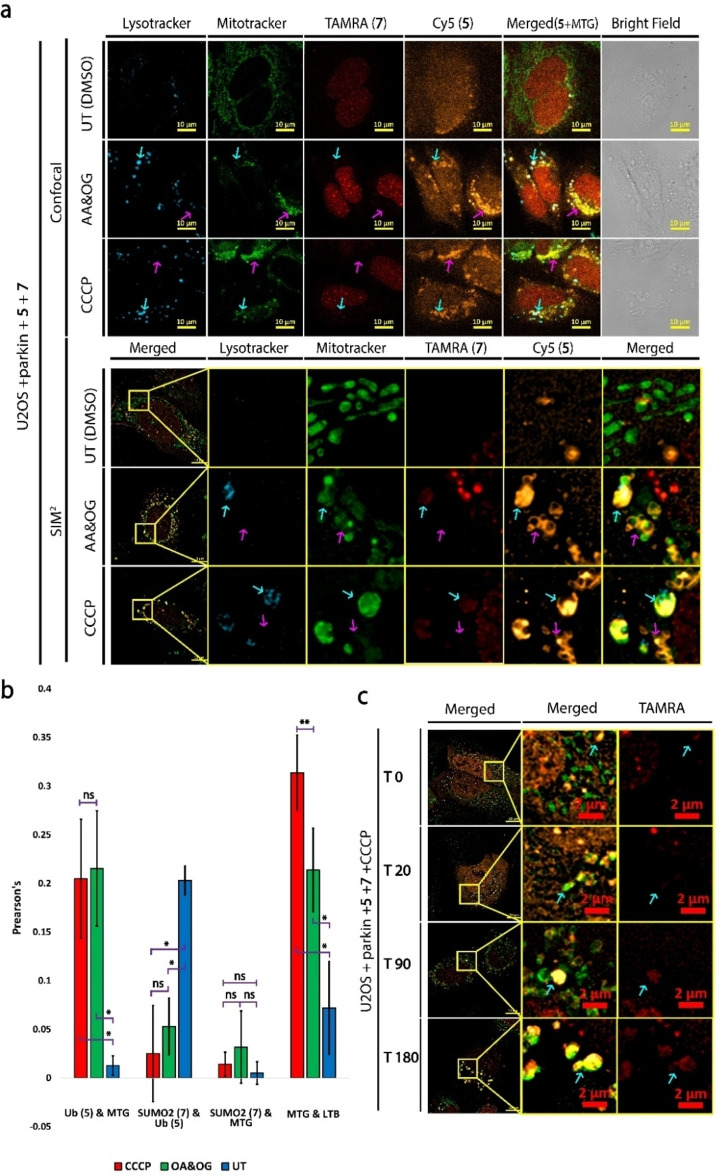
Live cell imaging of U2OS+parkin cells loaded with Ub **5** and SUMO2 **7** treated with CCCP, AA/OG and untreated (DMSO) with LTB and MTG staining. (a) Live cell LSCM and SIM^2^. Magenta arrow is perinuclear aggregates. Cyan arrow is lysosomes. (b) Colocalization analysis of cells from LSCM images in A, using averaged Pearson's coefficient from three independent experiments (>100 cells each). Probe **5** fluorescence was used for ROI except in the comparison of **7** and MTG colocalization where probe **7** was used for ROI. Scale bars are 10 μm. Error bars are standard deviation.* P‐Value is below 0.01, ** P‐Value is below 0.05, ns is not significant. (c) SIM^2^ time laps imaging during CCCP treatment of cells loaded with **5** and **7** and stained with MTG. Cyan arrow is lysosomes. Scale bars are 2 μm. Representative images of three independent experiments.

In our previous experiments, SUMO2 **7** colocalized with LTB but not SUMO2ΔG93 **9**. To directly compare the mitochondrial recruitment of SUMO2 to that of Ub in mitophagy, we loaded U2OS+parkin cells with Ub **5** and SUMO2 **7**. Following MBL, we treated the cells with either CCCP or a mixture of oligomycin (OG) and antimycin A (AA), as an alternative condition,^[50]^ for four hours and stained mitochondria with mitotracker green (MTG) and lysosomes with LTB. Live cell CLSM and SIM^2^ confirmed that Ub **5** is recruited to damaged mitochondria within four hours, while SUMO2 **7** was not detected (Figure 6a). Despite SUMO2’s **7** lysosomal recruitment, it mostly maintains its nuclear functions in contrast to the global rearrangement of Ub landscape. This observation is evident by the reduction in colocalization between Ub and SUMO2 during mitophagy (Figure 6b). Notably, although both CCCP and AA&OG treatments showed similar efficiencies in inducing mitophagy (Figure 6b), the latter was more toxic to cells, therefore we proceeded with CCCP only for the following experiments.

As a negative control, we performed the same experiment in U2OS wt and the +parkin cells. In U2OS cells, CCCP treatment resulted in fragmentation of the mitochondrial network. Nevertheless, this fragmentation did not result in localization of Ub probe **5** to mitochondria when compared to the +parkin cells (Supporting Information Figure S11). In these cells, we visualized a basal SUMO2 probe **7** localization to lysosomes that was similar to +parkin cells, suggesting that SUMO2 basal localization to lysosomes is not a parkin dependent process.

We then turned to visualize Ub and SUMO2 conjugation dynamics during mitophagy. For this, we loaded +parkin cells with Ub **5** and SUMO2 **7** followed by MTG staining and treated the cell with CCCP inside an incubated chamber. Time laps SIM^2^ images of these cells revealed that Ub **5**’s and SUMO2 **7**’s intensities increase in MTG positive vesicles after CCCP treatment (Figure 6c). Following depolarization, mitochondria fragments are pulled to the perinuclear area, where they are heavily ubiquitinated on the mitochondrial outer membrane (MOM) without SUMO recruitment. After 180 min from depolarization, larger MTG and LTB positive bodies started to appear and were positive for both Ub **5** and SUMO2 **7** (Figure 6c). In same cells without CCCP treatment, we did not observe any noticeable changes in the localization of either probe (Supporting Information Figure S12).

## Discussion

Studying the exceptional diversity and dynamics of PTMs is faced with many challenges. In addition, limited tools are available for these studies, which makes it harder to acquire knowledge for basic and translational science.^[51]^ For example, visualizing most PTMs is only possible by its indirect labeling *via* immunofluorescence approaches, most of which cannot be performed in living cells. Comparing the live cell localization and function of PTMs in general and of Ub as well as its related UBLs such as SUMO1‐3, in particular, is a notoriously challenging process.[^24^, ^52^] Therefore, developing additional tools to overcome these limitations is crucial for further understanding of these PTMs in particular and others in general.

Current methods to deliver “custom‐made proteins” suffer from various limitations such as the requirement of substantial cargo optimization to avoid endosomal entrapment.^[11]^ Previously, we found that while the delivery of Ub modified with Cyclic‐deca Arg was successful, the delivery of phosphorylated Ub failed and was successful only when using CPP modified with DABCYL.^[39]^ In addition, the requirement to prepare and attach the CPP often prolongs the synthesis, add further constrains and lead to losses of the precious “custom‐made” proteins.

In this study, we demonstrated the power of MBL for simultaneously delivering several synthetic proteins, without endosomal entrapment and CPP attachment. Our approach, which requires short incubation times (2 min), enables visualization of fluorescently labeled synthetic Ub and SUMO2 probes by directly loading them into cells with minimal interference to endogenous proteins. This allows instant and unprecedented control of the composition and fluorescent output of these protein probes in living cells, and to include a negative control in same cells. The number of delivered proteins is limited by the number of excitation lasers and available filters. Yet, without these constrains, MBL is in principle unlimited to the number of simultaneously delivered proteins paving the road for new ways to manipulate the composition of the cellular proteome. MBL introduces a new level of control into the notoriously dynamic and complex system of PTMs in living cells, independently of DNA transfection, and possibly allow rescuing knock out cells with loaded active enzymes.

**Figure 7:**
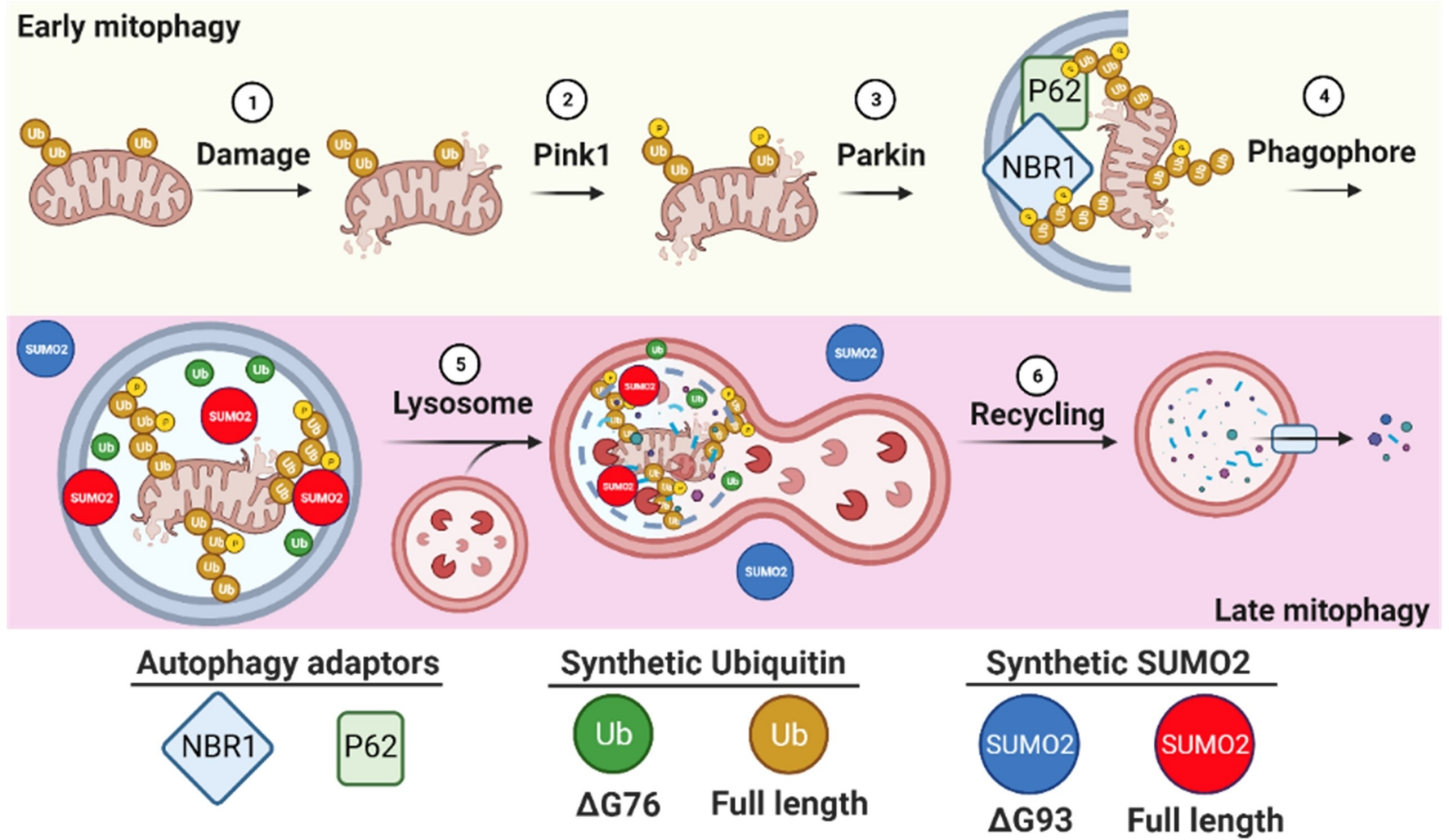
Schematic illustration of synthetic SUMO2, SUMO2ΔG93, Ub and UbΔG76 involvements during the different stages of early and late mitophagy.

By comparing Ub to its unconjugated form in same cells, we revealed that both proteins are localized to lysosome and autophagosomes during late mitophagy (Figure 7). It is unclear why the unconjugated Ub localizes also to this site, but this is possibly could be as a result of the interactions between free Ub and its receptors, which could regulate the ratio of autophagy and proteasomal degradation by altering the available Ub pools, as reported in the activation of P62 during “Ub stress”.^[53]^

Interestingly, while SUMO2 exhibited significant recruitment to lysosomes, SUMO2ΔG93 was not recruited, which suggests that SUMO2 is conjugated to lysosomal proteins. In addition, our results suggest that SUMO2 recruitment to lysosomes occurs in a late stage of mitophagy (Figure 7) and does not significantly affect its nuclear association with PML bodies. By comparing the localization of SUMO2 and SUMO2ΔG93 to nuclear puncta, we suggest that SUMO2 is recruited to PML bodies in live cells as a result of conjugation and not due to free multiple SUMOs interactions with other proteins *via* their SUMO interacting motifs. To best of our knowledge this is the first time that SUMO2 has been shown to be involved in lysosome, where its exact role still to be determined (Figure 7). A possible reason why such a function of SUMO2 has never observed by imaging approaches is that lysosomes can be disrupted upon cell fixation.^[42]^

SUMO2, the most important isoform in mammals,[^54^, ^55^] and SUMO3 share 97 % sequence identity with only 3 amino acids difference in their mature form. As a result, SUMO2 and SUMO3 lack specific antibodies, which makes it challenging to differentiate between the endogenous proteins in live or fixed cells. This is a serious limitation for our understanding of SUMO2/3 functions, since their sequence similarity is not fully reflected in their biochemical properties.^[28]^ Our method offers great opportunities to probe SUMO2/3 functions in live cells with isoform specificity in MLO dynamics without genetic manipulation.

## Conflict of interest

The authors have no competing interests to declare.

1

## Supporting information

As a service to our authors and readers, this journal provides supporting information supplied by the authors. Such materials are peer reviewed and may be re‐organized for online delivery, but are not copy‐edited or typeset. Technical support issues arising from supporting information (other than missing files) should be addressed to the authors.

Supporting Information

## Data Availability

The data that support the findings of this study are available in the supplementary material of this article.
