# Supplementary material for: Multiplexed Delivery of Synthetic (Un)Conjugatable Ubiquitin and SUMO2 Enables Simultaneous Monitoring of Their Localization and Function in Live Cells
Source: Chembiochem. 2022 Mar 11;23(11):e202200122. doi: 10.1002/cbic.202200122 (PMC9401080; doi:10.1002/cbic.202200122)
Supplement: Supplementary file 1 — Supporting Information [file CBIC-23-0-s001.pdf]

ChemBioChem

Supporting Information

Multiplexed Delivery of Synthetic (Un)Conjugatable Ubiquitin and SUMO2 Enables Simultaneous Monitoring of Their Localization and Function in Live Cells

Guy Mann[+], Pradeep Sadhu[+], and Ashraf Brik*

Table of Contents

[1]	General reagents	4
[2]	List of the protected amino acids used in peptides synthesis	4
[3]	List of cell culture reagents	5
[4]	List of antibodies for immunofluorescence	5
[5]	Fmoc-SPPS general procedure for 2-CTC resin	5
[6]	Fmoc-SPPS general procedure for rink amide resin	6
[7]	HPLC for peptide analysis and purification	6
[8]	General cell culture procedure	7
[9]	General procedure for establishing stable parkin over expression	8
[10]	General procedure for protein loading via multiplexed bead loading (MBL)	8
[11]	General procedure for CCCP and OA&OG mitophagy induction	8
[12]	General procedure for mitotracker, lysotracker and Hoechst staining	9
[13]	General method for confocal imaging and image analysis	9
[14]	General procedure for cell fixation and immunostaining	11
[15]	Dye labeling with maleimide fluorophores	11

[16] Synthesis Ub (1-76) analogues 12

[17] Synthesis of Ub (1-75) analogues 14

[18] Synthesis of SUMO-2-C48A (1-93) analogues 15

[19] Synthesis of SUMO-2-C48A (1-92) analogues 17

[20] Synthesis of FITC-PEG2-TAB2-ZnF4 18

[21] Live cell multiplex delivery of three Ub analogues and comparison between BL and MBL 20

[22] Live cell multiplex delivery of three different proteins 21

[23] Fluorescent gel analysis 21

[24] Live cell delivery of Cy5-Ub (**5**) and TAMRA-SUMO-2 (**7**) and mitophagy studies in fixed cells 22

[25] Live cell imaging of Cy5-Ub (**5**) and TAMRA-SUMO-2 (**7**) in U2OS ±parkin cells 25

[26] Time laps imaging of Cy5-Ub (**5**) and TAMRA-SUMO-2 (**7**) analogues without mitophagy induction 26

[27] References 27

1. General reagents:

Peptides were prepared by SPPS either manually in Teflon filter fitted syringes (purchased from Torviq) or by using an automated peptide synthesizer (CS336X, CSBIO). Analytical grade *N,N*-dimethylformamide (DMF) was purchased from Biotech. Resins were purchased from Creosalus, protected amino acids were purchased from GL Biochem and activating reagents N,N,N′,N′-Tetramethyl-O-(1H-benzotriazol-1-yl)uroniumhexafluorophosphate (HBTU), 1-Hydroxybenzotriazolemonohydrate (HOBt), O-(1H-6-Chlorobenzotriazole-1-yl)-1,1,3,3-tetramethyluronium hexafluorophosphate (HCTU), 1-[Bis(dimethylamino)methylene]-1H-1,2,3-triazolo[4,5-b]pyridinium 3-oxid hexafluorophosphate (HATU)] were purchased from Luxembourg Bio Technologies. Tetramethylrhodamine-5-maleimide received from anaspec. fluorescein-5-maleimide, Dylight 405 maleimide (DL405), Alexa Fluor 488 C5 maleimide (AF488) and pluronic F-68 were procured from thermofisher scientific. Cy5-maleimide from AAT Bioquest, {2[2-(Fmoc amino)ethoxy]ethoxy}acetic acid (PEG) was purchased from apollo scientific. Glass beads (<106 μm), PFA were purchased from sigma aldrich, 4-12% MOPS gel (MP41G12) was purchased from Merck. Additional miscellaneous chemicals were purchased from sigma aldrich, strem chemicals and alfa aesar.

2. List of the protected amino acids used in peptides synthesis:

Fmoc-Gly-OH, Fmoc-Ala-OH, Fmoc-Val-OH, Fmoc-Leu-OH, Fmoc-Ile-OH, Fmoc-Phe-OH, Fmoc-His(Trt)-OH, Fmoc-Asn(Trt)-OH, Fmoc-Gln(Trt)-OH, Fmoc-Arg(Pbf)-OH, Fmoc-Lys(Boc)-OH, Fmoc-Tyr(^{t}Bu)-OH, Fmoc-Ser(^{t}Bu)-OH, Fmoc-Thr(^{t}Bu)-OH, Fmoc-Asp(O^{t}Bu)-OH, Fmoc-Glu(O^{t}Bu)-OH, Fmoc-Lys(Alloc)-OH, Fmoc-Cys(Trt)-OH, Fmoc-Nle-OH, Fmoc-

Lue-Thr(ψMe,MePro)-OH, Fmoc-Ile-Thr(ψMe,MePro)-OH, Fmoc-Asp(OtBu)-(DMB)Gly-OH, Fmoc-Leu-Ser(ψMe,MePro)-OH and Fmoc-Asp-Thr($\psi^{Me,Me}$Pro)-OH

3. List of cell culture reagents:

Dulbecco's modified eagle's medium low glucose (DMEM-LG), phenol free DMEM (Opti-DMEM), fetal bovine serum (FBS), L-Gln, antibiotics (penicillin/streptomycin), trypsin/EDTA and phosphate buffered saline (PBS) were purchased from biological industries. Hoechst 33342 solution (20 mM), mitotracker green (L7526), mitotracker deep red (M22426) and lysotracker blue (L7525) probes were purchased from thermo-fisher. 8 well μ-Slide for live cell and 8 well chambered removable immunofluorescence slides for confocal microscopy were purchased from ibidi, 2-((3-chlorophenyl)hydrazono)malononitrile (CCCP) and poly-L-lysine hydro bromide was purchased from sigma.

4. List of antibodies for immunofluorescence:

Anti-phospho-Ub (Ser65), AF488 conjugate (ABS1513-I, sigma), PML (PA5-51039, thermos-fisher scientific), NBR1 (abnova, H00004077-M01), MTCO2 (abcam, ab110258), LAMP1 (santa cruz biotechnology, sc-20011). Secondary goat anti rabbit IgG, AF488 conjugate (abcam, ab150077), secondary goat anti mouse IgG, AF488 conjugate (abcam, ab150113), primary antibodies were stored by diluting in TRIS stabilizing solution (candor bioscience).

5. Fmoc-SPPS general procedure for 2-CTC resin:

2-Chlorotritylchloride (CTC) resin (0.7 mmol/g) was loaded with 0.6 eq. of Fmoc-Gly-OH and 2 eq. of DIEA in dry DCM for 1h. Following resin esterification, the Fmoc-Gly-resin was washed with DCM (X5), methanol (X5), and DMF (X5). The final loading was verified by Fmoc group absorbance after the DBU treatment to be 0.42 mmol/g. After initial resin loading, Fmoc-SPPS

was carried on automated peptide synthesizer (CS336X, CSBIO) in presence of 4 eq. of amino acid, 4 eq. of HCTU, and 8 eq. of DIEA to the initial loading of the resin for 50 min. Dipeptides were coupled manually using 2.5 eq. of amino acid, 2.5 eq. of HATU, and 5 eq. of DIEA to the initial loading of the resin for 1 h 30 min. To cleave the peptides from the solid support, the resin was washed with DMF (X5), MeOH (X5), DCM (X5), and dried under high vacuum. A cocktail of TFA:triisopropylsilane (TIS):H_2O (95:2.5:2.5) was added to resin and the reaction mixture was shaken for 2h at RT. The resin was filtered, and the combined filtrate was added dropwise to a 10-fold volume of cold ether and centrifuged. The precipitate was purged with nitrogen, dissolved in acetonitrile-water and freeze-drying in the lyophilizer to give the crude peptide.

6. Fmoc-SPPS general procedure on a Rink amide resin:

Fmoc-SPPS was carried on an automated peptide synthesizer (CS336X, CSBIO) in presence of 4 eq. of amino acid, 4 eq. of HCTU, and 8 eq. of DIEA to the initial loading of the resin for 50 min. The dipeptides were coupled manually using 2.5 eq. of amino acid, 2.5 eq. of HATU, and 5 eq. of DIEA to the initial loading of the resin for 1 h 30 min. To cleave the peptides from the solid support the resin was washed with DMF (X5), MeOH (X5), DCM (X5) and dried under high vacuum. A cocktail of TFA: triisopropyl silane (TIS): H_2O (95:2.5:2.5) was added to the resin and the reaction mixture was shaken for 2h at RT. The resin was filtered and the combined filtrate was added dropwise to a 10-fold volume of cold ether and centrifuged. The precipitate was purged with nitrogen, dissolved in acetonitrile-water for freeze-drying in the lyophilizer to give the crude peptide.

7. HPLC for peptide analysis and purification:

Analytical HPLC was performed on a thermo instrument (dionex ultimate 3000) using analytical column XSelect (thermo scientific hypersil gold, C18, 3 μm, 4.6 × 150 mm) at a flow rate of 1.2

mL/min and preparative HPLC was performed on a thermo instrument (dionex ultimate 3000) using preparative column XSelect (phenomenex jupiter, C18, 5 μm, 250 × 21.2 mm) at a flow rate of 15 mL/min. Semi-preparative HPLC was performed on a thermo instrument (dionex ultimate 3000) using chromatographie multohigh bio C18 10 μm, 250 × 10 mm column, at a flow rate of 4 mL/min. All synthetic products were purified by HPLC and characterized by mass spectrometry using the LCQ Fleet Ion Trap (thermo Scientific). All calculated masses have been reported as an average isotope composition. All HPLC steps were performed using a gradient from 0-60% buffer B over 30 min. Buffer A: 0.1% TFA in water; buffer B: 0.1% TFA in acetonitrile.

8. General cell culture procedure:

U2OS (HTB-96™ ATCC®S) cells were cultured in DMEM-low glucose supplemented with 10% FBS, 0.2 mM L-Gln and antibiotics (penicillin/streptomycin) in a humidified 37 °C incubator at 5% CO_2. Prior to imaging, culture medium was replaced with opti-DMEM-low glucose supplemented with 10% FBS, 0.2 mM L-Gln and antibiotics (penicillin/streptomycin). To detach cells from culture flasks, the media was aspirated and the flask was washed with sterile calcium and magnesium free PBS before cells were treated with 0.25% Trypsin + 0.02% EDTA solution and returned to incubation chamber for 5 min. Trypsin was quenched by adding the FBS supplemented media. The cell suspension was collected and the cells were pelleted (2 min at 1,000xg). Media was then aspirated and the cell pellet was re-suspended in fresh media. The cell density was determined using automated cells counter (Countess II, Invitrogen). For confocal microscopy, cells were seeded on poly-L-lysine (PLL) treated 8 well chamber slides (Ibidi) with removable silicone chamber in 3.4×10^4 *cells* per *well* and were allowed to reach ~90% confluence (24 h).

9. General procedure for establishing stable parkin over expression:

The retroviral plasmid pBMN-Parkin for overexpression of untagged human parkin was a gift from Dr. michael lazarou (Addgene plasmid repository #89299). U2OS cells stably expressing parkin were produced as previously described.[1]

10. General procedure for protein loading *via* multiplexed bead loading (MBL):

Cells were cultured in 8 well ibdi chamber slides to 90% confluence. The protein constructs were diluted from concentrated 1000X DMSO stocks into sterile PBS with 0.1% pluronic® F-68. The protein concentration was determined by Bradford assay against a BSA calibration curve and the concentrations were adjusted to a final of 9 µM. For MBL, equal volumes of each construct were mixed prior to cell treatment. For loading, the culture medium was removed and cells were washed once with warm PBS+0.1% pluronic® F-68 solution. To the washed cells, 150µl of the protein solution was added (to cover the cells) and a monolayer of glass beads (<106 µm, Sigma Aldrich) was sprinkled over the cells. The culture slide was tapped on bench eight times, with gently swirling the slide for a total of two times in between tapping, and incubated for 2 min at 37 °C under 5% CO_2. Following incubation, the cells were washed with DMEM (0.3 mL X 2) and incubated for 30-60 min at 37 °C under 5% CO_2 with opti-DMEM to recover from the loading process for a duration of 30 min.[2]

11. General procedure for CCCP and OA/OG mitophagy induction:

The peptide delivery was performed according to general procedure and after the medium was aspirated and cells were washed with PBS (0.3 mL x 1), treated with 20 µM CCCP in Full-

DMEM (0.3 mL) or 5 μM of oligomycin A + 5 μM Antimycin A in Full-DMEM (0.3 mL) and incubated for 4 h at 37 °C under 5% CO_2.

12. General procedure for mitotracker, lysotracker and Hoechst staining:

Cells were washed with PBS (0.3 mL x 2), and treated with 150 nM of mitotracker and 250 nM of lysotracker, in serum free DMEM and incubated for 15 min 37 °C under 5% CO_2. Cells were washed with PBS (0.3 mL x 2) and kept throughout imaging process in 0.3 mL of opti-DMEM with or without 1 μg/mL of Hoechst.

13. General method for imaging and image analysis:

Distribution of fluorescent peptides in live cells was analyzed using a laser scanning confocal microscopy (LSCM) (Confocal Zeiss LSM 710, Axio observer) equipped with C-Apochromate X40/NA1.2 water immersion objective for live cell imaging and a Plan-Apochromat X63/NA 1.4 oil immersion lens for fixed samples. All channels were adjusted to 0.8 AU pinhole settings with 3.5 μs pixel dwell. Four lasers were used for the different tags; UV laser (Hoechst, DAPI, Lysotracker blue, DyLight405) – 405 nm (10 mW), Argon multi-line laser (Mitotracker green, AF488, FITC) – 454, 488 and 514 nm (30 mW), Green laser (TAMRA) – 543 nm (10 mW) – Red laser (Cy5, AF637, AF647) – 639 nm (5 mW). During live cell CLSM analysis, the samples were maintained at 37 °C in a humidified chamber. Fixed cells were imaged at room temperature. All the imaging parameters were determined in the preliminary experiments containing unstained and single stained controls.

Single representative images were analyzed using ZEN 3.0 (ZEN-lite) software. All channels were normalized to uniform intensity by using the range indicator tool (using a representative image) and all adjustments were identically applied to all the images of the different treatments.

Co-localization analysis was performed on all unprocessed images collected (> 100 cells per experimental condition, ~10 images per condition) using FiJi software. The analysis was performed using a self-generated macro file using the coloc 2 plugin with Otsu dark auto threshold under the relevant masks to produce the average pearson's coefficient for each image, these were used to generate a coefficient for each biological replicate. Presented coefficients are average of averages from three independent experiments. Error bars are reported as standard deviation of the average of averages.

Super resolution microscopy (SRM) was performed using structure illuminated matrix (SIM) on a Zeiss Lattice SIM (Andor1, AxioObserver) using C-Apochromate X63/NA1.2 water immersion objective for live cell imaging and a Plan-Apochromat X63/NA 1.46 oil immersion lens for fixed samples. Four lasers were used for the different tags; UV diode laser (Hoechst, DAPI, Lysotracker blue) – 405 nm (0.5% of 50mW), Blue diode laser (Mitotracker green, AF488) – 488 (2 % of 500mW) Green diode laser (TAMRA) – 561 nm (2 % of 500mW) – Red diode laser (Cy5, AF647) – 642 nm (0.5% of 500mW). During live cell CLSM analysis, the samples were maintained at 37 °C in a humidified chamber. Fixed cells were imaged at room temperature. All the imaging parameters were determined in the preliminary experiments containing unstained and single stained controls.

SRM images were processed in ZEN black 3.0 by SIM^2 algorithm using default parameters (Leap, Low SNR Input, 16 iterations). All channels were normalized to uniform intensity by using the range indicator tool, using a representative image, and all adjustments were identically applied to images of different conditions to avoid data manipulation. Non-linear signal enhancement was applied to SIM^2 images to compensate for low intensity; any modification was identically applied to all images from the different treatments.

14. General procedure for cell fixation and immunostaining

After the indicated treatments, the cells in ibdi 8 well chamber slides were gently washed twice with warm PBS followed by fixation with warm 4% Paraformaldehyde (w/v in PBS) for 20 minutes in dark at room temperature (RT). The cells were again washed thrice with PBS and then permeabilized with 1% Triton X-100 for 5 minutes in dark at RT. Blocking was done with 5% BSA (w/v in PBS supplemented with 0.02% sodium azide) for 1 h in dark at RT. The cells then were incubated with specific primary antibodies at manufacturer recommended dilutions for either 1 h at RT or overnight at 4°C, followed by 3 washes with PBST (PBS with 0.02% Tween-20) and then incubated with appropriate fluorophore-conjugated secondary antibodies (abcam) for 1 h at RT in dark. The cells were again washed thrice with PBST. Following the completion of treatments, cells were immersed in Fluoromount G (Invitrogen) either with or without DAPI. The slides were stored at -20 °C until imaging.

15. Dye labeling with maleimide fluorophores

The sulfhydryl-containing protein was dissolved in 6M guanidine hydrochloride (Gn.HCl)/200 mM phosphate buffer pH = 7.3 (2 mM) and cooled to 0 °C. A solution of maleimide conjugated fluorophore (1.2 equiv, dissolved to 10 mM in DMF) was added to the sulfhydryl-containing protein solution and allow the reaction to proceed for 2-4 hours at 0 °C.[3] The reaction was monitored by analytical HPLC (C18 column), using a gradient of 0-60% B over 30 min. Finally, the crude peptide was purified using a C18 semi-preparative column with the gradient flow of 0-

60% B over 30 min and purified labeled protein was isolated and lyophilized. Dry purified proteins were dissolved in DMSO to X1000 stock and kept at -20 °C.

16. Synthesis of Ub (1-76) analogues:

(a) Sequence:

C-PEG-PEG-

NleQIFVKT**LT**GKT**IT**LEVEPSDTIENV

KAKIQDKEGIPPDQQRLIFAGKQLE

DGRT**LS**DYNIQKESTLHLVLRLRGG

(b)

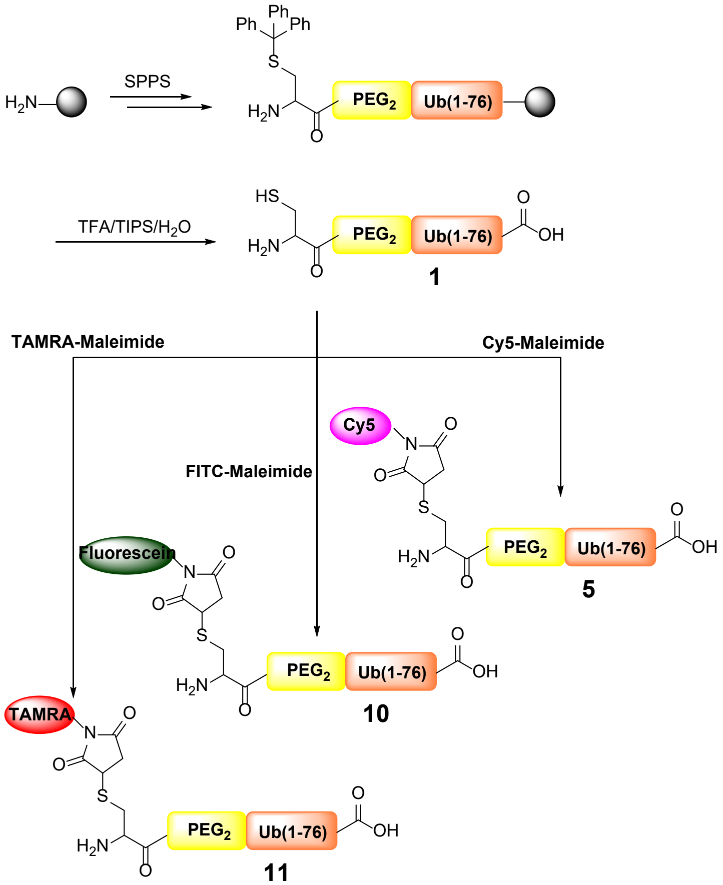

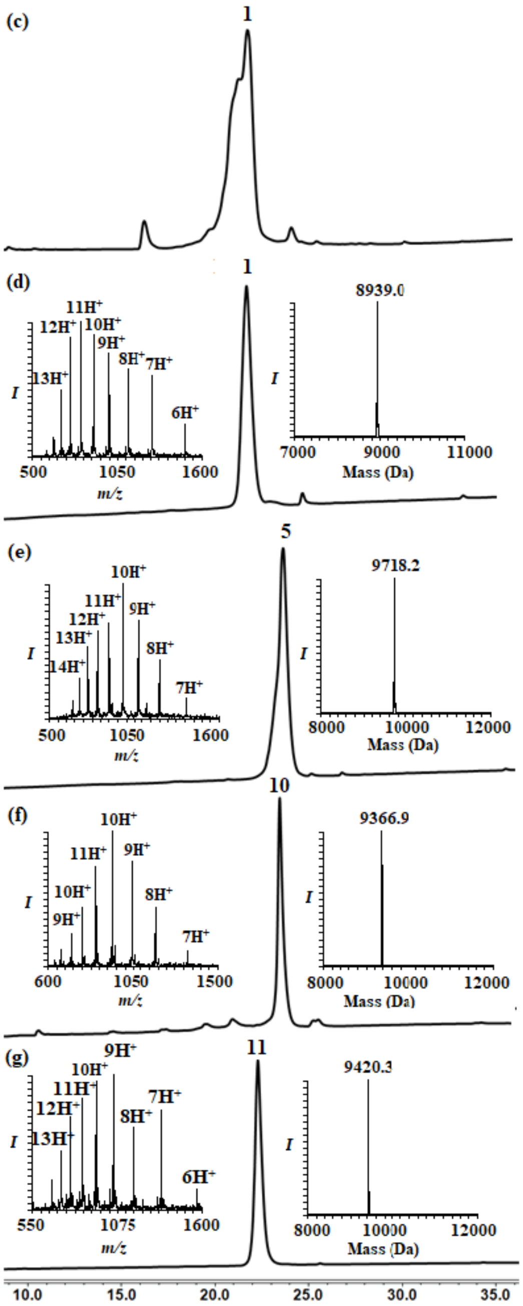

Figure S1. Synthesis of Cys-PEG$_2$-Ub-COOH and its labelled conjugates: (a) Sequence of Ub. (b) Schematic representation of synthetic route for **5, 10** and **11**. (c) Analytical HPLC chromatogram of crude **1** (detection at 214 nm). (d) Analytical HPLC and mass analysis of purified **1** with the observed mass 8939 ± 0.7 Da, (calcd 8939.9 Da) (average isotopes). (e) Analytical HPLC and mass analysis of purified **5** with the observed mass 9718.2 ± 0.6 Da, (calcd 9717.8 Da) (average isotopes). (f) Analytical HPLC and mass analysis of purified **10** with the observed mass 9366.9 ± 1.0 Da, (calcd 9367.3 Da) (average isotopes). (g) Analytical HPLC and mass analysis of purified **11** with the observed mass 9420.3 ± 1.5 Da, calcd 9421.4 Da (average isotopes).

Cys-PEG$_2$-Ub(1-76)-COOH **(1)**, was prepared on pre-swollen 2-Chlorotrityle chloride resin (0.1 mmol) pre-loaded with Fmoc-Gly (as described in the general procedure). Fmoc amino acids were coupled on an automated peptide synthesizer using 4 equiv amino acid, 4 equiv HCTU and 8 equiv of DIEA. Pseudoproline dipeptides Fmoc-Lue-Thr(ψMe,MePro)-OH, Fmoc-Ile-Thr(ψMe,MePro)-OH, Fmoc-Asp(OtBu)-(DMB)Gly-OH and Fmoc-Leu-Ser(ψMe,MePro)-OH were coupled manually at indicated positions (highlighted in the sequence in **Figure S1a** using 2.5 eq. of dipeptide, 2.5 eq. of HATU and 5 eq. of DIEA for 1.5 h and the linker [2-[2-(Fmoc-amino)ethoxy]ethoxy]acetic acid (abbreviated as PEG) was coupled manually using 4 eq. amino acid, 4 eq. HATU and 8 eq. DIEA for 1.5 h to afford **1**. The crude peptide **1** was purified by preparative HPLC using C18 column with a gradient of 0-60% buffer B over 30 min to afford the corresponding peptide Cys-PEG$_2$-Ub(1- 76)-COOH **(1)** in ~19 % isolated yield. The isolated product was labelled with Cy5-maleimide, TAMRA-maleimide and Fluorescein-maleimide as described in the general procedures to obtain the **5**, **10** and **11**, respectively.

17. Synthesis of Ub(1-75) analogues:

(a) Sequence:

C-PEG2-

NleQIFVKT**LT**GKT**IT**LEVEPSDTIEN

VKAKIQDKEGIPPDQQRLIFAGKQL

E**DG**RT**LS**DYNIQKESTLHLVLRLRG

(b)

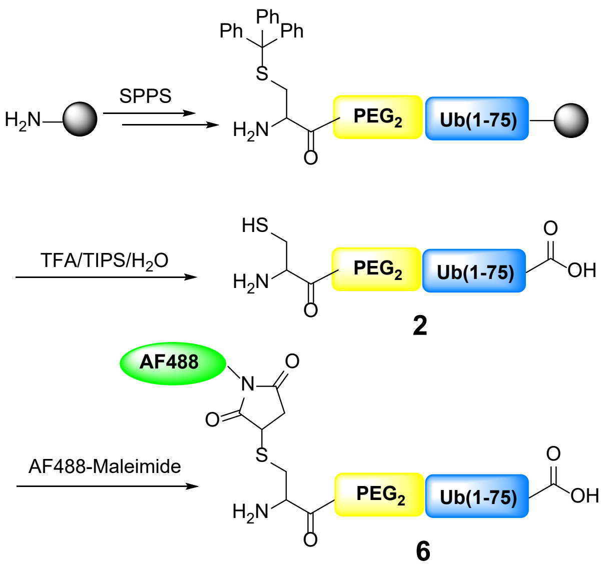

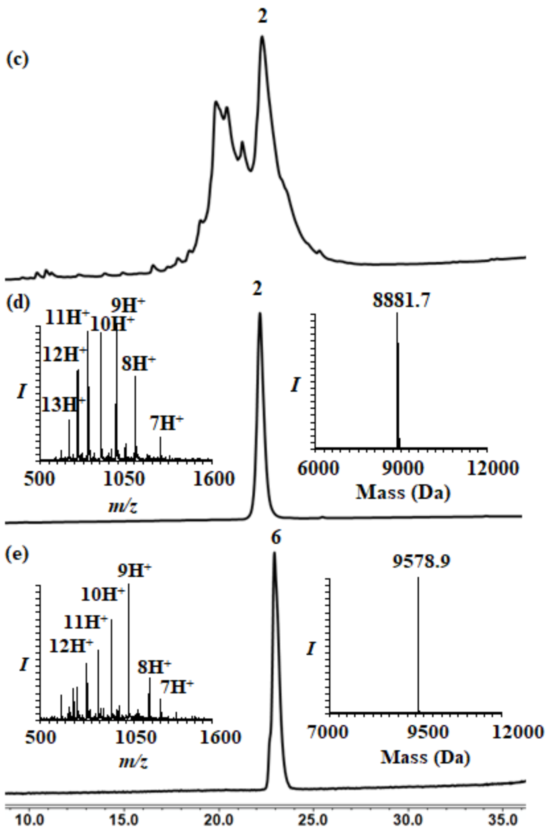

Figure S2. Synthesis of Cys-PEG-PEG-Ub(1-75)-COOH conjugates: (a) Sequence of Ub. (b) Schematic representation of synthetic route for **6**. (c) Analytical HPLC chromatogram of crude **2** (detection at 214 nm). (d) Analytical HPLC and mass analysis of purified **2** with the observed mass 8881.7 ± 0.5 Da, calcd 8882.9 Da (average isotopes). (e) Analytical HPLC and mass analysis of purified **6** with the observed mass 9578.9 ± 0.5 Da, calcd 95879.9 Da (average isotopes).

Cys-PEG2-Ub(1-75)-COOH (**2**), was prepared on pre-swollen 2-Chlorotrityle chloride resin (0.1 mmol) pre-loaded with Fmoc-Gly (as described in the general procedure, and section 16). The crude peptide **2** was purified by preparative HPLC using C4 column with a gradient of 0-60% buffer B over 30 min to afford the corresponding peptide Cys-PEG-PEG-Ub(1- 75)-COOH **(2)** in

~18 % isolated yield. The isolated product were labelled with AF488-maleimide as described in the general procedures and isolated the targeted product **6**.

18. Synthesis of SUMO-2-C48A (1-93) analogues:

(a) Sequence:

C-PEG-PEG-

NleADEKKEGVKTENNDHINLKVAGQ**DG**S

VVQFKIKRHTP**LS**KLNlesKAYAERQG**LS**Nle

RQIRFRF**DG**QPINETDTPAQLENleEDE**DT**ID

VFQQQTGG

(b)

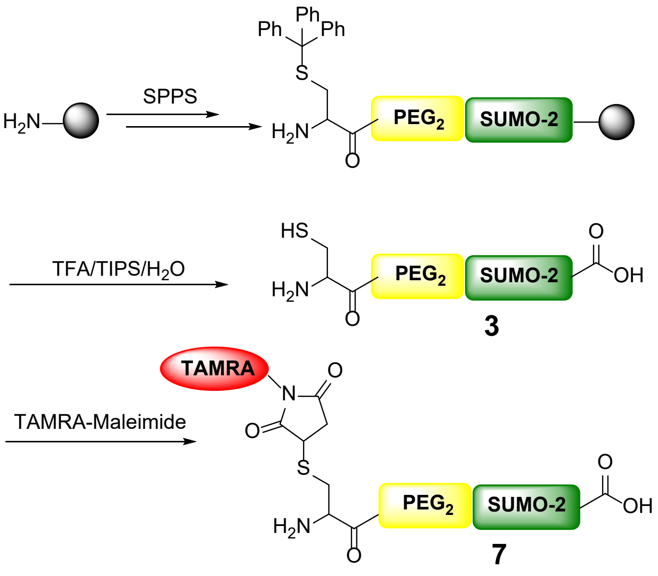

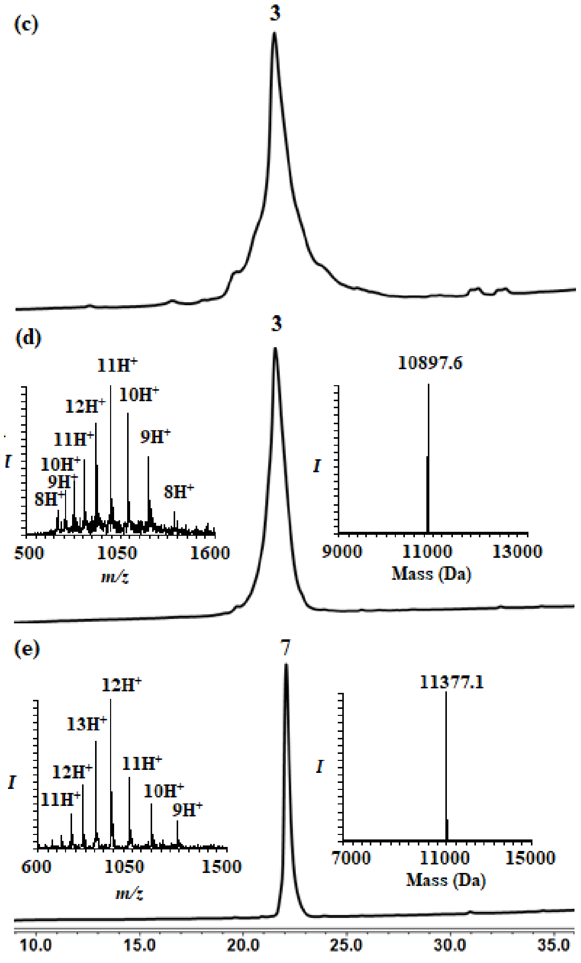

Figure S3. Synthesis of Cys-PEG2 -SUMO-2(1-93)-COOH and its conjugates: (a) Sequence of SUMO-2. (b) Schematic representation of synthetic route for **7**. (c) Analytical HPLC chromatogram of crude **3** (detection at 214 nm). (d) Analytical HPLC and mass analysis of purified **3** with the observed mass 10897.6 ± 1.7 Da, calcd 10897.8 Da (average isotopes). (e) Analytical HPLC and mass analysis of purified **7** with the observed mass 11377.1 ± 0.6 Da, calcd 11379.3 Da (average isotopes).

Cys-PEG_2-SUMO-2(1-93)-COOH **(3)**, was prepared on pre-swollen 2-Chlorotrityle chloride resin (0.1 mmol) pre-loaded with Fmoc-Gly (as described in the general procedure). Fmoc amino acids were coupled on an automated peptide synthesizer using 4 equiv amino acid, 4 equiv HCTU and 8 equiv of DIEA. Pseudoproline dipeptides Fmoc- Asp(OtBu)-(DMB)Gly-OH, Fmoc-Asp-Thr($\psi^{Me,Me}$pro)-OH and Fmoc-Leu-Ser($\psi^{Me,Me}$pro)-OH were coupled manually at indicated positions shown in **Figure S3a** using 2.5 eq. of dipeptide, 2.5 eq. of HATU and 5 eq. of DIEA for 1.5 h and the linker [2-[2-(Fmoc-amino)ethoxy]ethoxy]acetic acid (abbreviated as PEG) was coupled manually using 4 eq. amino acid, 4 eq. HATU and 8 eq. DIEA for 1.5 h to afford **3**. The cysteine at position 48 was mutated to alanine. The crude peptide **3** was purified by preparative HPLC using C4 column with a gradient of 0-60% buffer B over 30 min to afford the corresponding peptide Cys-PEG-PEG-SUMO-2(1-93)-COOH **(3)** in ~21 % isolated yield. The isolated Sulfhydryl-containing product was labelled with TAMRA-maleimide as described in the general procedures to yield the desired product **7**.

19. Synthesis of SUMO-2-C48A (1-92) analogues:

(a) Sequence:

C-PEG2-

NleADEKKEGVKTENNDHINLKVAGQ**DG**S

VVQFKIKRHTP**LS**KLNleKAYAERQG**LS**Nle

RQIRFRF**DG**QPINETDTPAQLENleEDE**DT**ID

VFQQQTG

(b)

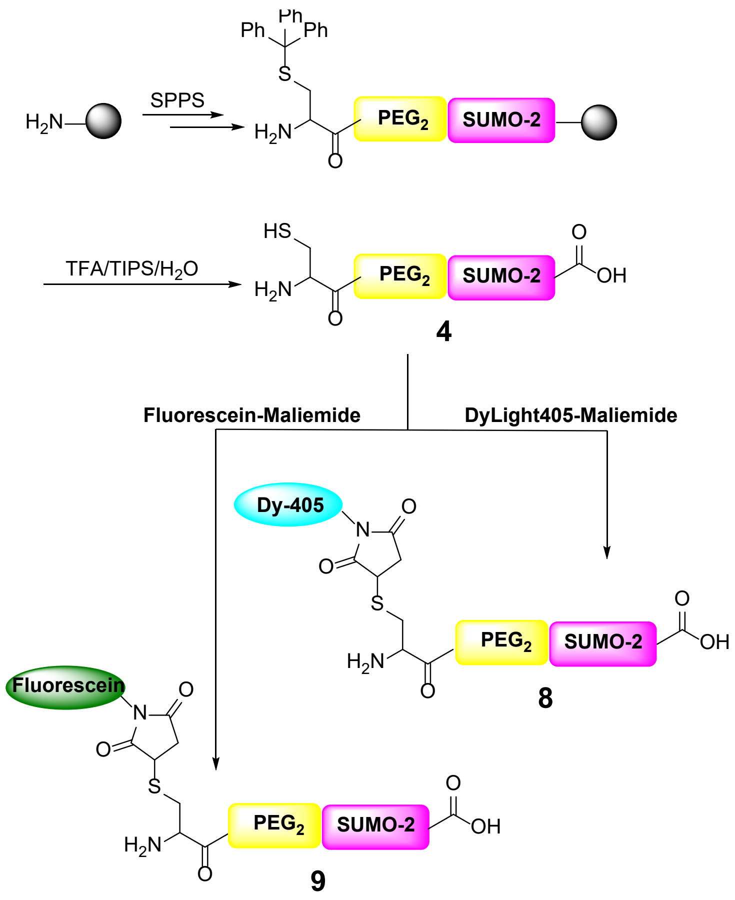

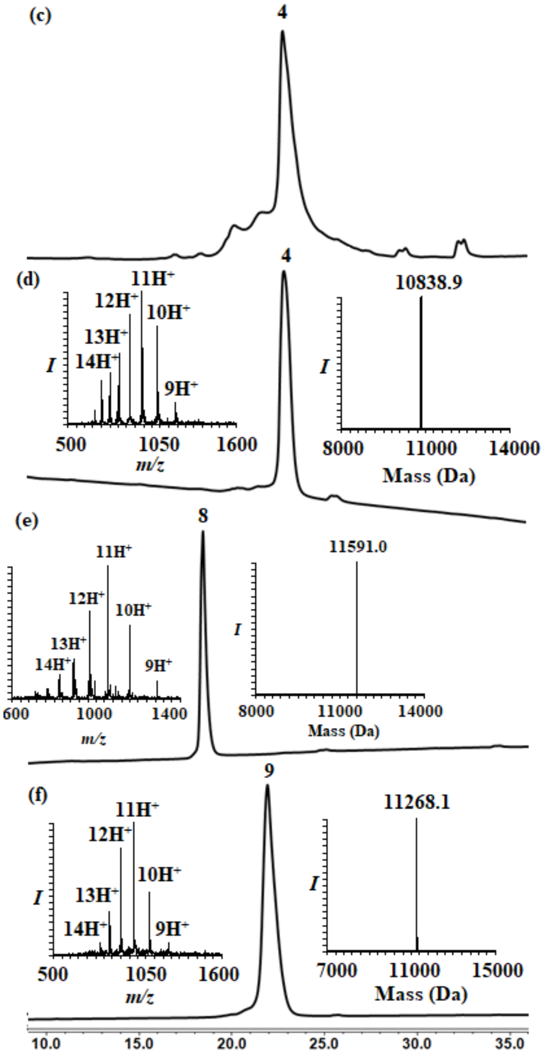

Figure S4. Synthesis of Cys-PEG$_2$-SUMO-2(1-92)-COOH conjugates: (a) Sequence of SUMO-2. (b) Schematic representation of synthetic route for **8, 9**. (c) Analytical HPLC chromatogram of crude **4** (detection at 214 nm). (d) Analytical HPLC and mass analysis of purified **4** with the observed mass 10838.9 ± 0.5 Da, calcd 10840.8 Da (average isotopes). (e) Analytical HPLC and mass analysis of purified **8** with the observed mass 11591.0 ± 0.1 Da. (f) Analytical HPLC and mass analysis of purified **9** with the observed mass 11268.1 ± 1.2 Da, calcd 11268.1 Da (average isotopes).

Cys-PEG$_2$-SUMO-2(1-92)-COOH **(4)**, was prepared on pre-swollen 2-Chlorotrityle chloride resin (0.1 mmol) pre-loaded with Fmoc-Gly (as described in the general procedure, and section 18). The crude peptide **3** was purified by preparative HPLC using C4 column with a gradient of 0-60% buffer B over 30 min to afford the corresponding peptide Cys-PEG-PEG-SUMO-2(1-92)-COOH **(4)** in ~23 % isolated yield. The isolated product was labelled with Dylight 405-maleimide and Fluorescein-maleimide as described in the general procedures to yield the products **8**, **9** respectively.

20. Synthesis of FITC-PEG$_2$-TAB2-ZnF4:

(a) Sequence:

FITC-PEG$_2$-

DDEGAQWNCTACTFLNHPALIRCEQCEMPRHF

(b)

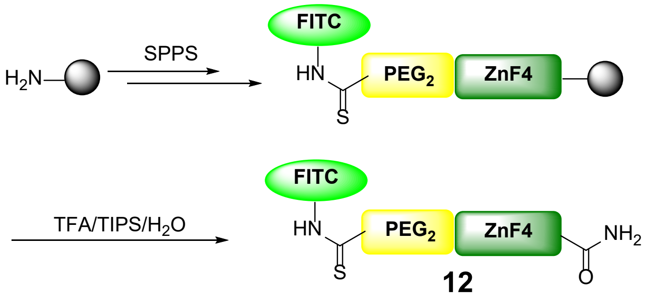

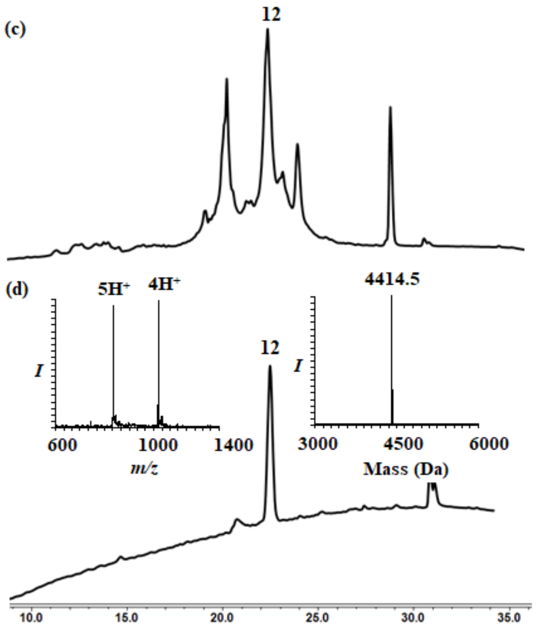

Figure S5. Synthesis of FITC-PEG$_2$-TAB2-ZnF4 -CONH$_2$: a, Sequence of FITC-PEG$_2$- TAB2-ZnF4 -CONH$_2$. b, Schematic representation of synthetic route for **12**. c, Analytical HPLC chromatogram of crude **12** (detection at 214 nm). (d) Analytical HPLC and mass analysis of purified **12** with the observed mass 4414.5 ± 0.7 Da, calcd 4415.6 Da (average isotopes).

FITC-PEG$_2$-TAB2-ZnF4 **(12)**, was prepared on pre-swollen rink amide resin (0.1 mmol) as described in the general procedure to give FITC-PEG$_2$- TAB2-ZnF4 **(12)**. The crude peptide **12** was purified by preparative HPLC using C18 column with a gradient of 0-60% buffer B over 30 min to afford the corresponding peptide FITC-PEG$_2$-TAB2-ZnF4 **(12)** in ~26 % isolated yield.

21. Live cell multiplex delivery of three Ub analogues and comparison between BL and MBL:

Ub analogues labelled with Cy5 (**5**), TAMRA (**10**) and Fluorescein (**11**) were delivered along with mix (**5** + **10** + **11**) into the U2OS cells and treated with Hoechst before imaging as described in the general procedure (Figure 6 a). We compared BL and MBL using EGFP, **5** and **7** in same cells with Hoechst staining (Figure 6b).

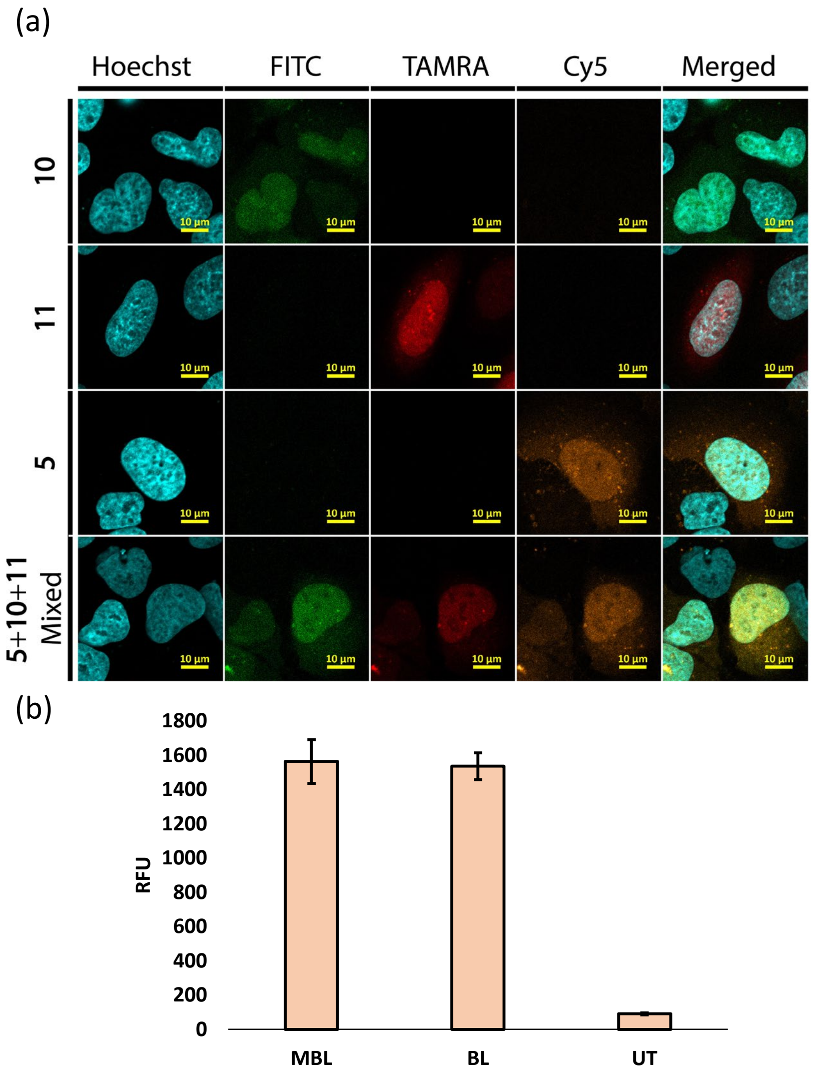

Figure S6. MBL and BL of Ub and SUMO2 analogues. (a) Representative live cell images of Fluorescein labelled Ub (**10**), TAMRA labelled Ub (**11**), Cy5 labelled Ub (**5**), and mixed (**5** + **10** + **11**). Scale bars are 10µm. (b) Comparison of nuclear TAMRA intensity between MBL (EGFP, **5** and **7**), BL (only **7**) and untreated cells. Error bars are standard deviation.

22. Live cell MBL of three different proteins:

Three different proteins labelled with fluorophores of different emission wavelength such as Ub labelled with Cy5, SUMO-2 labelled with TAMRA and TAB2-ZnF4 labelled with FITC were

delivered separately as well as mix (**5** + **7** + **12**) into the U2OS wild type cells and treated with Hoechst before imaging as described in the general procedure.

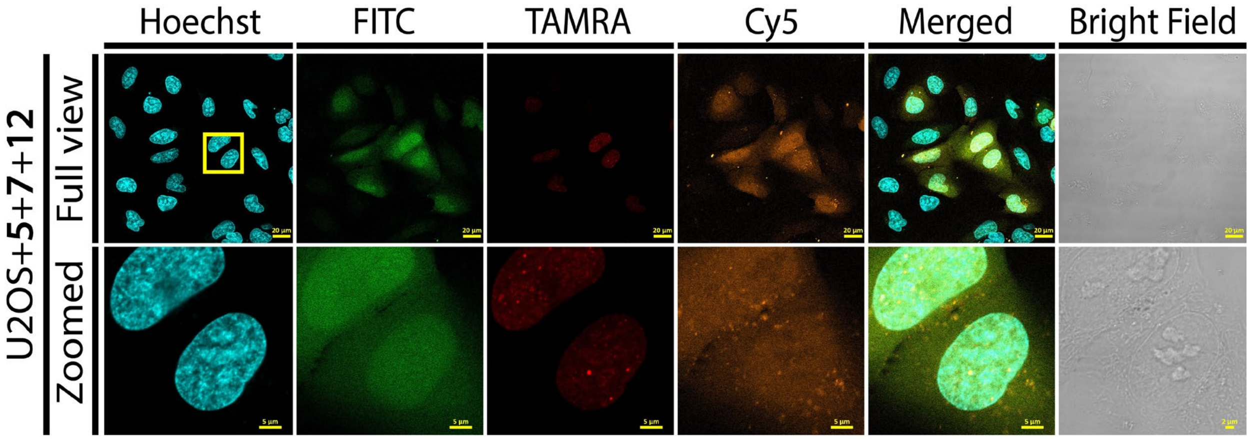

Figure S7. Representative live cell images of Cy5 labelled Ub (**5**), TAMRA labelled SUMO-2 (**7**) and FITC labelled TAB2-ZnF4 (**12**) and mixed (**5** + **7** + **12**) loaded *via* MBL. Scale bars are 20 µm for full view and 5 µm for zoomed.

23. Fluorescent gel analysis:

For SDS-PAGE analysis of three-protein delivery, the proteins **5**, **7** and **10** were mixed and loaded into U2OS cell cultured on an 8-well plate (according to the general procedure). Following the loading procedure, cells were incubated for 60 min at 37 °C under 5% CO_2 and then treated with and without 10 µm of MG-132 in full-DMEM for overnight. Then the medium was aspirated and washed with (PBS X 2) and lysed with Fluorescent reducing buffer and heated for 5 min at 95 °C and ran the gel using 4-12% MOPS gel. The gel was imaged using Fusion FX6 ECL and fluorescent detection system.

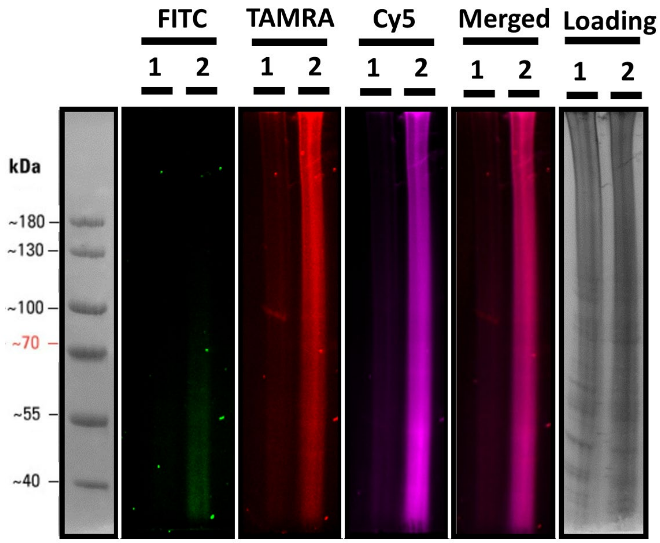

Figure S8. Fluorescent gel analysis using 4-12% MOPS gel with lysates from U2OS cells **5**, **7**, **12** proteins treated with and without MG-132, In each fluorophore line 1 represents the mixed lysate and line 2 represents the mixed lysate treated with 10 µm of MG-132 for overnight.

24. Live cell delivery of Cy5-Ub (5) and TAMRA-SUMO-2 (7) and mitophagy studies in fixed cells

To understand the function of Cy5-Ub (**5**) and TAMRA-SUMO-2 (**7**), these proteins were delivered together into the live U2OS +parkin cells and induced mitophagy by treating for 4 h with CCCP (**Figure S9a**). Cells were fixed and stained with antibodies for phospho-Ub (Ser65) conjugated with AF488 (1:500), MTCO2 (1:150), LAMP1 (1:100) and followed by labelling with AF488 conjugated secondary antibody (1:1000), as described in general procedure and imaged by SIM[2].

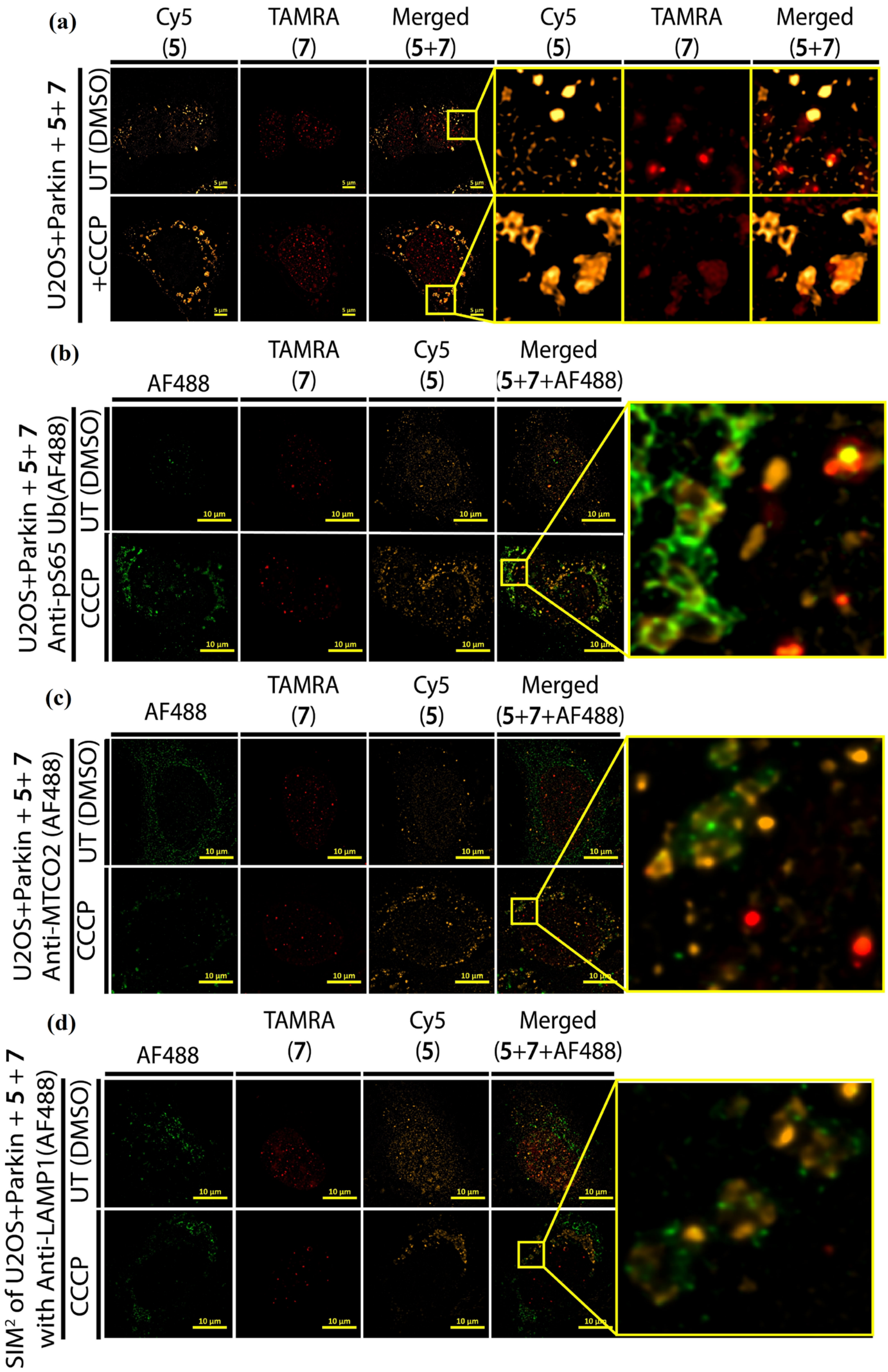

Figure S9. Representative SIM^2 images of U2OS + parkin, with ±CCCP for 4 h. (a) Zoomed images of fixed cells. (b) Zoomed images of fixed cells stained with phospho-Ub (Ser65)and labelled with Secondary antibody, AF488 conjugate. (c) Zoomed images of fixed cells stained with anti-MTCO2 and labelled with Secondary antibody, AF488 conjugate. (d) Zoomed images

of fixed cells stained with anti-LAMP1 and labelled with AF488 conjugate secondary antibody. Scale bars are 10µm.

Cells were washed with PBS X 2 and proceeded for fixation procedure (as described in the general procedure). Fixed cells were stained with Anti-NBR1 (1:150) followed by labelling with secondary antibody, AF488 conjugate (1:1000). Cells were imaged in both laser scanning confocal microscopy (LSCM) and super resolution microscopy via structure illuminated matrix (SIM^2).

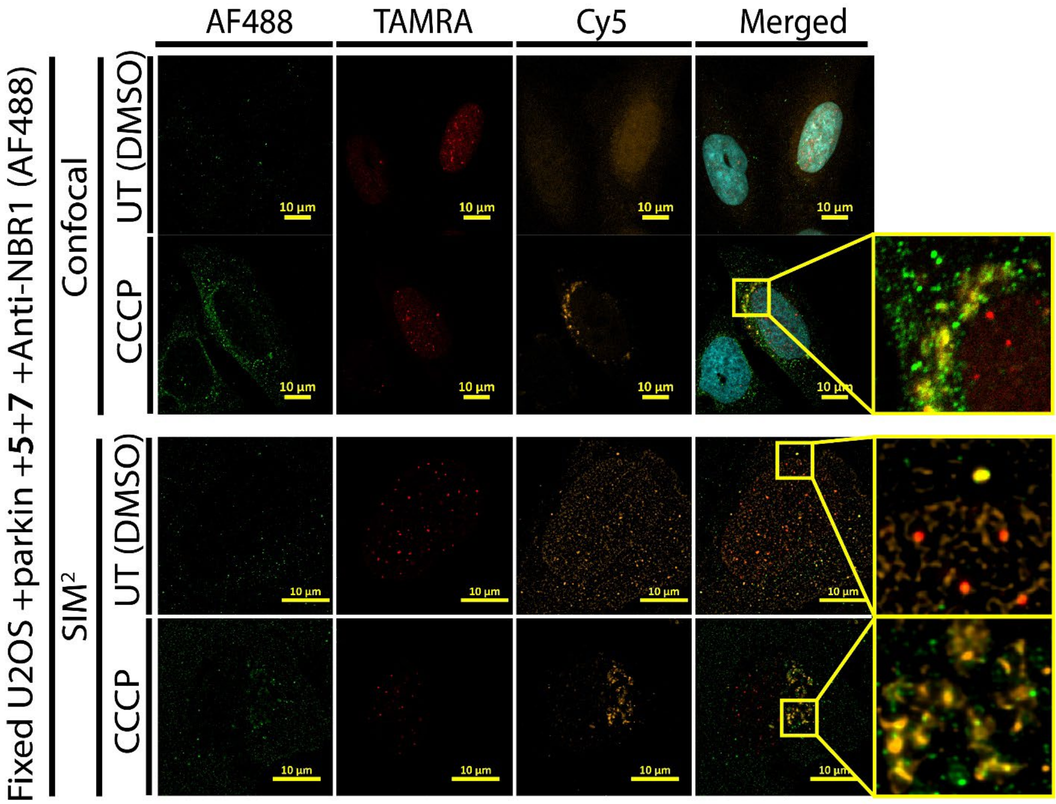

Figure S10. Representative CLSM and SIM^2 images of U2OS +parkin, treated with ±CCCP for 4 h and stained with Anti-NBR1. Scale bars are 10µm.

25. Live cell imaging of Cy5-Ub (5) and TAMRA-SUMO-2 (7) in U2OS ±parkin cells

To measure the involvement of Ub (**5**) and sumo-2 (**7**) analogues in mitophagy, we loaded **5** + **7** into live cells, with and without parkin expression, *via* MBL and induced mitophagy using 20 μM CCCP for 4 h. Following CCCP treatment, the cells were treated with lysotracker-blue and mitotracker green as described in the general procedure.

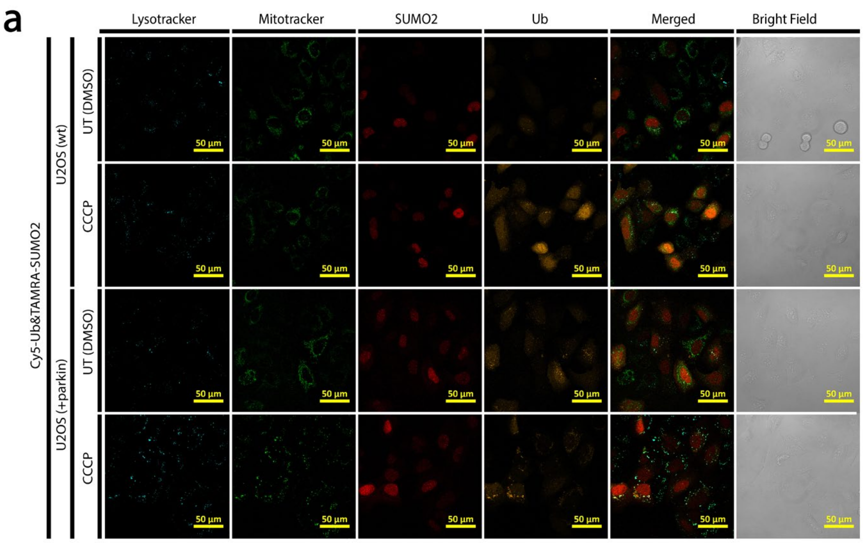

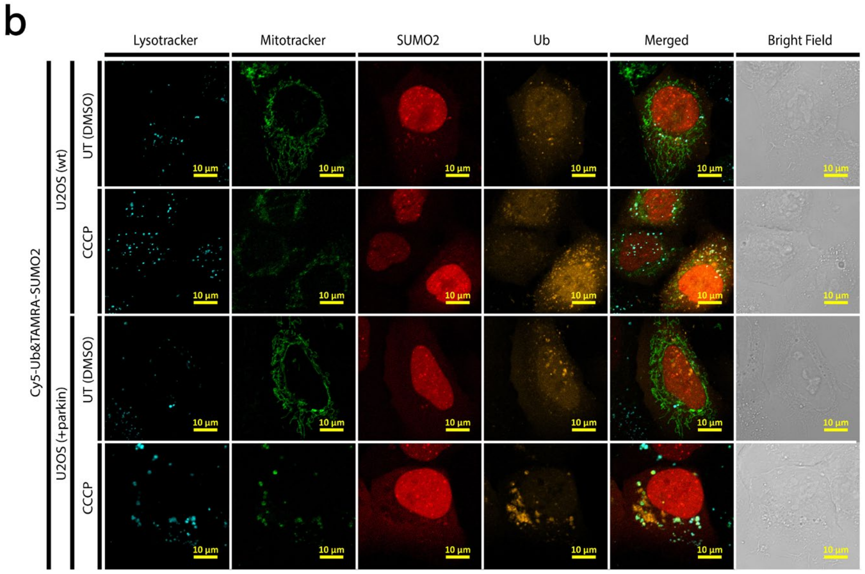

c

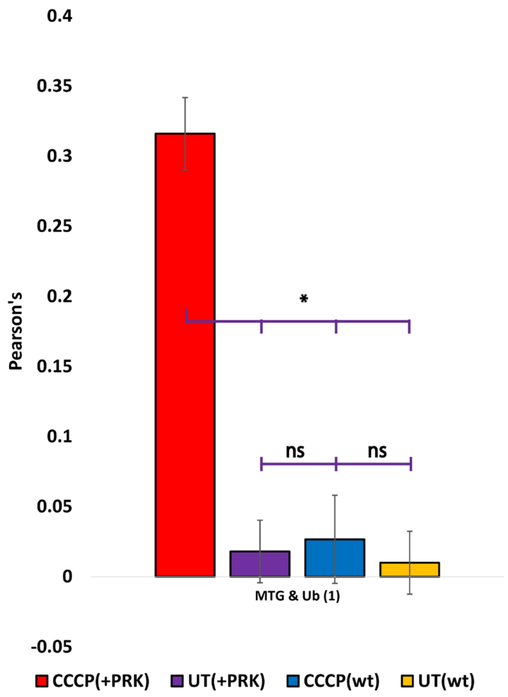

Figure S11. CLSM images of live U2OS, U2OS + parkin cells treated with ±CCCP for 4 h and stained with lysotracker-blue and mitotracker-green. (a) Full view images of **5&7** delivery in live cells. Scale bars are 50µm. (b) Zoomed images images of **5&7** delivery in live cells. Scale bars are 10µm. (c) Co-localization analysis of mitotracker-green and Ub (**5**), U2OS cells with and without parkin, treated with ±CCCP. Error bars are standard deviation.

26. Time laps imaging of Cy5-Ub (5) and TAMRA-SUMO-2 (7) analogues without mitophagy induction

To visualize the conjugation dynamics of Cy5-Ub (**5**) and TAMRA-SUMO-2 (**7**) in mitophagy. We loaded them into the U2OS + parkin cells and treated with and without CCCP for 4 h and stained with mitotracker green and imaged at various time points.

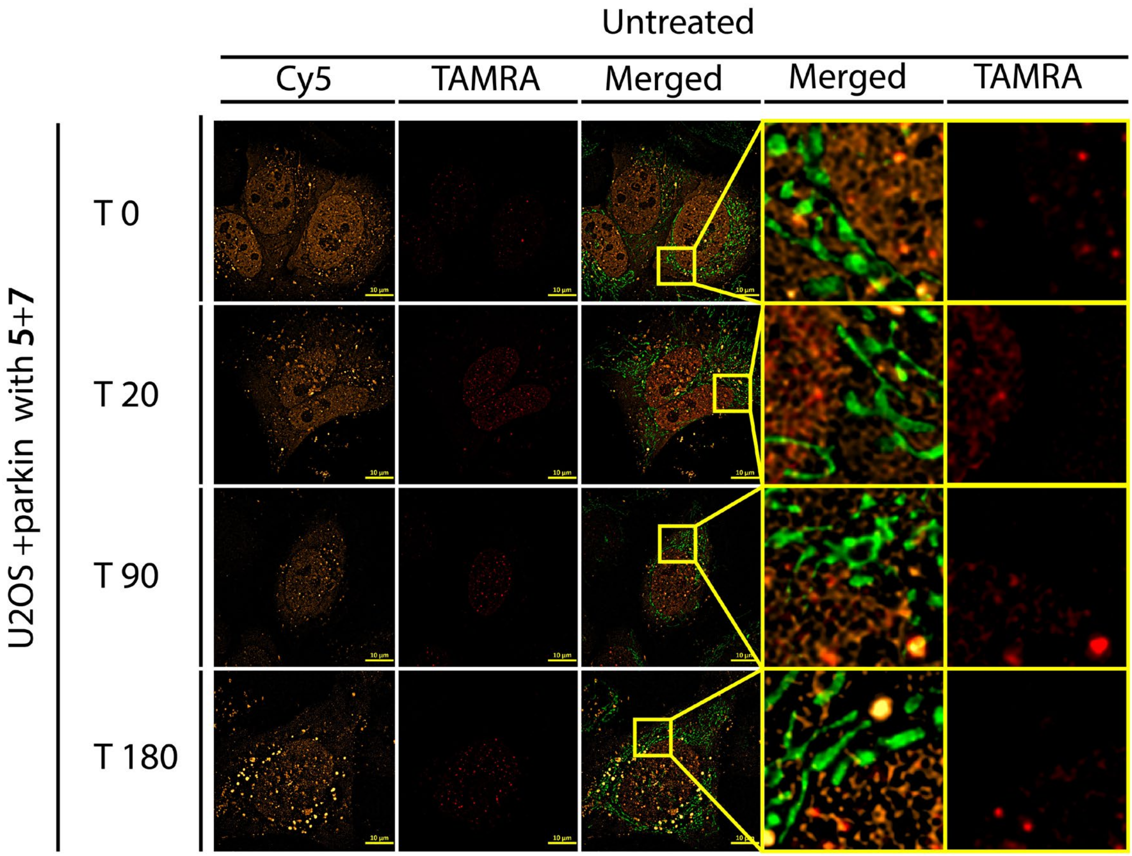

Figure S12. Representative zoomed SIM^2 images of U2OS +parkin cells loaded with **5** and **7** without CCCP treatment at different time points. Scale bars are 10µm.

References

[1] G. Mann, G. Satish, P. Sulkshane, S. Mandal, M. H. Glickman, Brik, A,. *Chem. Commun*. **2021,** *57*, 9438-9441.

[2] M. Bhagawati, S. Hoffmann, K. S. Höffgen, J. Piehler, K. B. Busch, H. D. Mootz, *Angew. Chem. Int. Ed.* **2020,** *132*, 21193 –21201.

[3] G. B. Vamisetti, S. Gandhesiri, P. Sulkshane, G. Mann, M. H. Glickman, A. Brik, *J. Am. Chem. Soc.* **2020**, *142*, 19558−19569.
